# Succession of the multi-site microbiome along pancreatic ductal adenocarcinoma tumorigenesis

**DOI:** 10.3389/fimmu.2024.1487242

**Published:** 2024-11-07

**Authors:** Yiqing Zhu, Xiao Liang, Mengfan Zhi, Lixiang Li, Guoming Zhang, Changxu Chen, Limei Wang, Peng Wang, Ning Zhong, Qiang Feng, Zhen Li

**Affiliations:** ^1^ Department of Gastroenterology, Qilu Hospital of Shandong University, Jinan, Shandong, China; ^2^ Shandong Provincial Clinical Research Center for Digestive Disease, Qilu Hospital of Shandong University, Jinan, Shandong, China; ^3^ Laboratory of Translational Gastroenterology, Qilu Hospital of Shandong University, Jinan, Shandong, China; ^4^ Robot Engineering Laboratory for Precise Diagnosis and Therapy of Gastrointestinal (GI) Tumor, Qilu Hospital of Shandong University, Jinan, Shandong, China; ^5^ Shandong Key Laboratory of Oral Tissue Regeneration, Department of Human Microbiome, School and Hospital of Stomatology, Cheeloo College of Medicine, Shandong University, Jinan, Shandong, China; ^6^ Shandong Engineering Laboratory for Dental Materials and Oral Tissue Regeneration & Shandong Provincial Clinical Research Center for Oral Diseases, Shandong University, Jinan, Shandong, China

**Keywords:** pancreatic neoplasms, pancreatitis, endoscopic ultrasound-guided fine needle aspiration, microbiota, time series analysis

## Abstract

**Background:**

To investigate microbial characteristics across multibody sites from chronic pancreatitis (CP), through pancreatic benign tumors, to pancreatic ductal adenocarcinoma (PDAC) at different stages.

**Methods:**

16S ribosomal RNA (rRNA) amplicon sequencing was conducted on saliva, duodenal fluid, and pancreatic tissue obtained via endoscopic ultrasound-guided fine needle aspiration (EUS-FNA) of patients with CP, pancreatic benign tumors, PDAC in stage I/II, III, and IV. The neutral community model (NCM) assessed the microbial assembly contribution and MaAslin2 identified the differential microbes.

**Results:**

From CP to stage IV PDAC patients, there was a marked surge in influence of salivary and duodenal microbiota on constitution of pancreatic microbial communities. Our observations revealed a successive alteration in microbial species across various bodily sites during PDAC tumorigenesis. Notably, *Porphyromonas gingivalis*, *Treponema denticola*, *Peptoanaerobacter stomatis*, *Propionibacterium acidifaciens*, *Porphyromonas endodontalis*, *Filifactor alocis*, etc., sequentially increased along PDAC progression in pancreatic tissue, whereas bacteria commonly used as probiotics *Bifidobacterium breve*, *Lactiplantibacillus plantarum*, etc., declined. Furthermore, the sequentially escalating trends of *Peptoanaerobacter stomatis* and *Propionibacterium acidifaciens* during PDAC tumorigenesis were mirrored in duodenal fluid and saliva. *Porphyromonas gingivalis*, *Porphyromonas endodontalis*, and *Filifactor alocis*, which intensified from CP to stage IV PDAC in pancreatic tissue, were also found to be enriched in saliva of patients with short-term survival (STS) compared with those with long-term survival (LTS).

**Conclusions:**

Salivary and duodenal microorganisms were prominent factors in shaping pancreatic microbial landscape in PDAC context. Further exploration of these microbial entities is imperative to unravel their specific roles in PDAC pathogenesis, potentially yielding insights for future therapeutic strategies.

## Introduction

Pancreatic cancer, predominantly pancreatic ductal adenocarcinoma (PDAC), is one of the most lethal diseases worldwide. PDAC ranks as the sixth leading cause of cancer-related death in China (1). Due to non-specific early symptoms and a lack of effective screening methods, approximately 80-85% of patients have entered an unresectable or metastatic stage at the time of diagnosis (2), with a 5-year survival rate of only 7.2% (3). This underscores the urgent need to elucidate the pathogenesis of PDAC and identify novel therapeutic targets.

Microbiota residing in diverse anatomical locales, encompassing the oral cavity, duodenum, intestines, and pancreas, among others, have been documented to correlate with various clinical markers indicative of PDAC (4–7). Specifically, the abundance of *Porphyromonas gingivalis* and *Aggregatibacter actinomycetemcomitans* in salivary fluids is associated with an elevated risk of PDAC (8). Furthermore, *Faecalibacterium prausnitzii* levels are frequently diminished in the saliva and feces of PDAC patients (6, 9), whereas patients experiencing long-term survival (LTS) display elevated concentrations of *Akkermansia muciniphila* and *Faecalibacterium prausnitzii* within the gut microbiome (10). In addition, the intra-tumoral microbiota (IM) within the pancreas plays a pivotal role in PDAC pathogenesis, potentially modulating the progression of the disease (11, 12). *Gammaproteobacteria* found in PDAC tumors can render the chemotherapeutic agent gemcitabine inactive, thereby contributing to chemotherapy resistance (7).

Furthermore, the human microbiota is inherently dynamic, encompassing fluctuations across short/long timescales (13). Numerous research endeavors have aimed to identify correlations linking temporal shifts in microbiota composition to diseases, notably type I diabetes, necrotizing enterocolitis, and infections (14). While investigations have illuminated disparities in gene expression patterns, metabolic signatures, and therapeutic responses among pancreatic precursor lesions, early-stage, and late-stage PDAC patients (15–18), the intricate microbial dynamics throughout diverse anatomical sites and stages of PDAC remain elusive. This gap in understanding impedes a definitive assessment of whether the distinctive microbial profiles observed between PDAC patients and controls serve as causal agents or merely consequential factors in the pathogenesis of PDAC.

The present study aimed to conduct a comprehensive analysis of the temporal dynamics of the oral, duodenal, and pancreatic microbial communities across pancreatitis, pancreatic benign tumors, and various stages of PDAC. Furthermore, we delved into identifying microbial signatures potentially involved in the initiation and progression of PDAC, thereby providing novel perspectives on its underlying mechanisms and potential therapeutic avenues.

## Methods

### Subject recruitment

We performed an observational study of 81 PDAC patients who underwent endoscopic ultrasound-guided fine needle aspiration (EUS-FNA) enrolled from January 2022 to December 2022 at the Qilu Hospital of Shandong University. The research adhered strictly to the Declaration of Helsinki principles and secured ethical clearance from the Ethics Committee of the Qilu Hospital, Shandong University, under reference KYLL-202111-018-1. Prior to enrollment, written informed consent was obtained from each participant. Eligible subjects included individuals aged over 18 years with suspected pancreatic lesions necessitating EUS-FNA for diagnostic confirmation.

The exclusion criteria were meticulously defined, encompassing: (I) concurrent presence of other cancers; (II) individuals who had undergone EUS-FNA, endoscopic retrograde cholangiopancreatography (ERCP), X-ray guided punctures, pancreatic surgeries, or received chemotherapy, radiotherapy, or other biological treatments within the preceding period; and (III) recent users (within the past month) of proton-pump inhibitors (PPIs), antibiotics (administered orally or intravenously), probiotics, prebiotics, or synbiotics. For clinicopathological staging, adherence was maintained to the 8th edition of the American Joint Committee on Cancer’s (AJCC) TNM staging system for PDAC. Additionally, PDAC patients were categorized into two distinct groups based on their chemotherapy response: the complete response/partial response (CR/PR) group comprised patients exhibiting complete disappearance of target lesions or a ≥30% reduction in the sum of the longest diameters of baseline lesions following treatment, while the stable disease/progressive disease (SD/PD) group encompassed those whose lesions either decreased slightly yet did not meet PR criteria, increased but fell short of PD status, demonstrated a ≥20% increase in the sum of longest lesion diameters, or manifested new lesions post-chemotherapy. Furthermore, regarding survival outcomes, PDAC patients were differentiated into short-term survival (STS) and LTS groups at the 16-month survival analysis point.

### Sample collection and processing

All participants underwent rinsing of their mouths with normal saline, adhering strictly to established protocols (19). Following this, the rinse was discarded, and a volume of 3 to 5 milliliters of saliva was promptly collected into a cryopreserved tube. The EUS-FNA procedure, performed by a seasoned endoscopist, utilized linear array echo endoscopes specifically from PENTAX (models EG-3870UTK and EG-3270UK, Tokyo, Japan). As the endoscope navigated to the descending segment of the duodenum, the aspirator delicately drew 5 milliliters of duodenal fluid into a sterile collector, which was then transferred to a cryopreserved tube. Prior to performing the color Doppler-guided puncture, a thorough examination of pancreatic lesions was conducted to ascertain the absence of major blood vessels along the intended needle path. Utilizing a 22-gauge needle sourced from COOK (USA), pancreatic tissue was meticulously collected and deposited into a sterile, cryopreserved tube. Immediately thereafter, all samples underwent rapid freezing in liquid nitrogen, were safely conveyed within liquid nitrogen containers, and stored in lock-in freezer chambers maintained at -80°C until the time of DNA extraction.

### DNA extraction and 16S rRNA amplicon sequencing

All samples underwent thawing on ice followed by aliquoting, whereupon microbial DNA extraction was performed utilizing the E.Z.N.A.^®^ soil DNA Kit sourced from Omega Bio-tek, Norcross, Georgia, USA. The DNA extract’s quality was verified both via 1% agarose gel electrophoresis and the NanoDrop 2000 UV-vis spectrophotometer from Thermo Scientific, Wilmington, USA. Subsequently, the hypervariable V3-V4 region of the bacterial 16S rRNA gene was amplified employing primer pairs 338F (5’-ACTCCTACGGGAGGCAGCAG-3’) and 806R (5’-GGACTACHVGGGTWTCTAAT-3’), facilitated by an ABI GeneAmp^®^ 9700 PCR thermocycler based in California, USA. The purified amplification products were then sequenced on the advanced Illumina MiSeq PE300 or NovaSeq PE250 platform, both originating from Illumina, San Diego, USA.

Paired-end reads were consolidated into sequence tags, adhering to their overlapping characteristics and subject to rigorous quality control measures executed by the UPARSE pipeline. This step excluded subpar reads and chimeric sequences, leveraging vsearch software (with parameters set to maxee = 3 and minlength = 370) for effective filtration. Upon dereplication, the remaining clean reads were clustered into distinct operational taxonomy units (OTUs) using a 97% similarity threshold. Representative reads from 7219 OTUs were aligned against the Ribosomal Database Project, version 18, to ascertain taxonomic identities, requiring an 80% similarity match to species entries within the reference database, thereby providing insights into the microbiome composition.

### Statistical analyses

We ran ‘Decontam’ (20) in order to identify and remove contaminant DNA sequences from the dataset. RStudio (R version 4.1.1) was employed for statistical analysis. Adonis2 was used to explore the correlation between the clinical indices and the microbiota. The alpha and beta diversity distance matrices were calculated using the “Vegan” package (version vegan_2.6-4). The Sloan neutral model was used to predict the relationship between occurrence frequency and relative abundance of species (21). The FEAST analysis was used to track the potential sources of the microbial structure (22). Microbial differences between groups were tested using MaAsLin2 analysis (version 2_1.14.1), and we adjusted the effects of age, gender, and body mass index (BMI). Genera or species with a *p*-value less than 0.05 were retained. At each position, we took the union of the differential microbes between the chronic pancreatitis (CP) group and each of the other four groups and applied the Mfuzz algorithm to cluster the expression trends of the average relative abundance of the differential microbes. According to the similarity of their abundant-variation patterns from CP to stage IV PDAC patients, the differential microbes were divided into four different clusters and named C1-C4. R package ‘ClusterGVis’ was used to generate the fitted trend from CP to stage IV PDAC patients for each cluster. Spearman correlation analysis was employed to establish the correlation network within each module (|Spearman correlation| > 0.6, false discovery rate (FDR)-adjusted p-value < 0.05), as well as to determine the interrelationships between clusters in different groups (|Spearman correlation| > 0.6, p-value < 0.01).

## Results

### Microbial composition in the multibody sites along the development of PDAC

**Figure 1:**
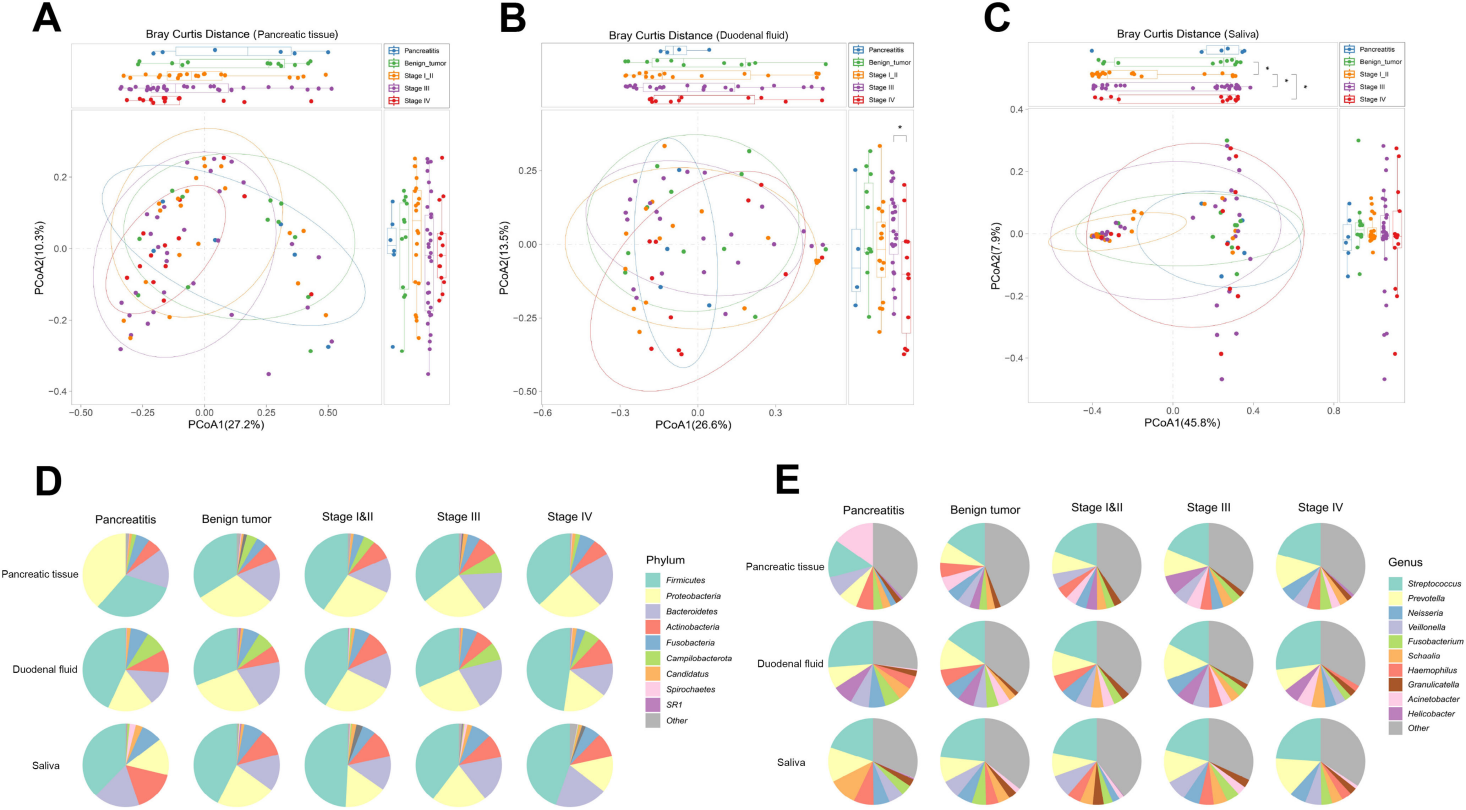
Microbial composition across CP, pancreatic benign tumors, PDAC in stage I/II, III, and IV groups in the multibody sites. **(A–C)** The principal co-ordinates analysis (PCoA) analysis was used to evaluate the microbial composition in pancreatic tissue **(A)**, duodenal fluid **(B)**, and saliva **(C)** across the five groups. **(D, E)** The microbial relative abundance across the five groups of the multibody sites at the phylum **(D)** and genus **(E)** levels.

We enrolled 81 subjects, of whom 6 had CP, 12 had pancreatic benign tumors including serous cystic neoplasm (SCN), mucinous cystic neoplasm (MCN), intraductal papillary mucinous neoplasm (IPMN), and solid pseudopaillary neoplasm (SPN), 20 had PDAC in stage I or II, 30 had PDAC in stage III, and 13 had PDAC in stage IV. Except for carbohydrate antigen 19–9 (CA 19-9), no significant statistical difference was observed in baseline characteristics among the five groups (**Supplementary Table S1**). We first measured the microbial alpha-diversity among the pancreatic tissue, duodenal fluid, and saliva of the five groups using different methodologies and found there was no significant difference (**Supplementary Figure S1**). We performed the beta-diversity analysis to compare the microbial composition. Clustering was detected for the Bray-Curtis principal co-ordinates analysis (PCoA) analysis among the five groups, especially in the saliva (**Figures 1A–C**).

In the pancreatic tissue, duodenal fluid, and saliva of the five groups, the predominant phyla were *Firmicutes*, *Proteobacteria*, *Bacteroidetes*, and *Actinobacteria*, demonstrating a broad phyla-level similarity (**Figure 1D**). However, differences appeared at the genus level (**Figure 1E**). In the pancreatic tissue, *Streptococcus* and *Prevotella* increased sequentially from CP, to benign tumors, to stage I/II PDAC, to stage III PDAC, finally to stage IV PDAC patients, while *Acinetobacter*, *Veillonella*, and *Haemophilus* decreased sequentially. *Helicobacter* showed an increasing tendency from CP to stage III PDAC patients. In the duodenal fluid, *Fusobacterium* reduced sequentially from CP to stage IV PDAC patients. Meanwhile, in the saliva, *Prevotella* also increased sequentially from benign tumors to stage IV PDAC patients.

### Succession of microbes in the multibody sites along the development of PDAC

To figure out the microbes that varied significantly along the development of PDAC, MaAsLin2 analysis was conducted to test the difference of microbes among the five groups. A total of 66, 66, and 72 microbial species in the pancreatic tissue, duodenal fluid, and saliva were identified, respectively. At the genus level, we identified 51, 62, and 46 differently distributed genera in the pancreatic tissue, duodenal fluid, and saliva, as shown in **Supplementary Figure S2**.

**Figure 2:**
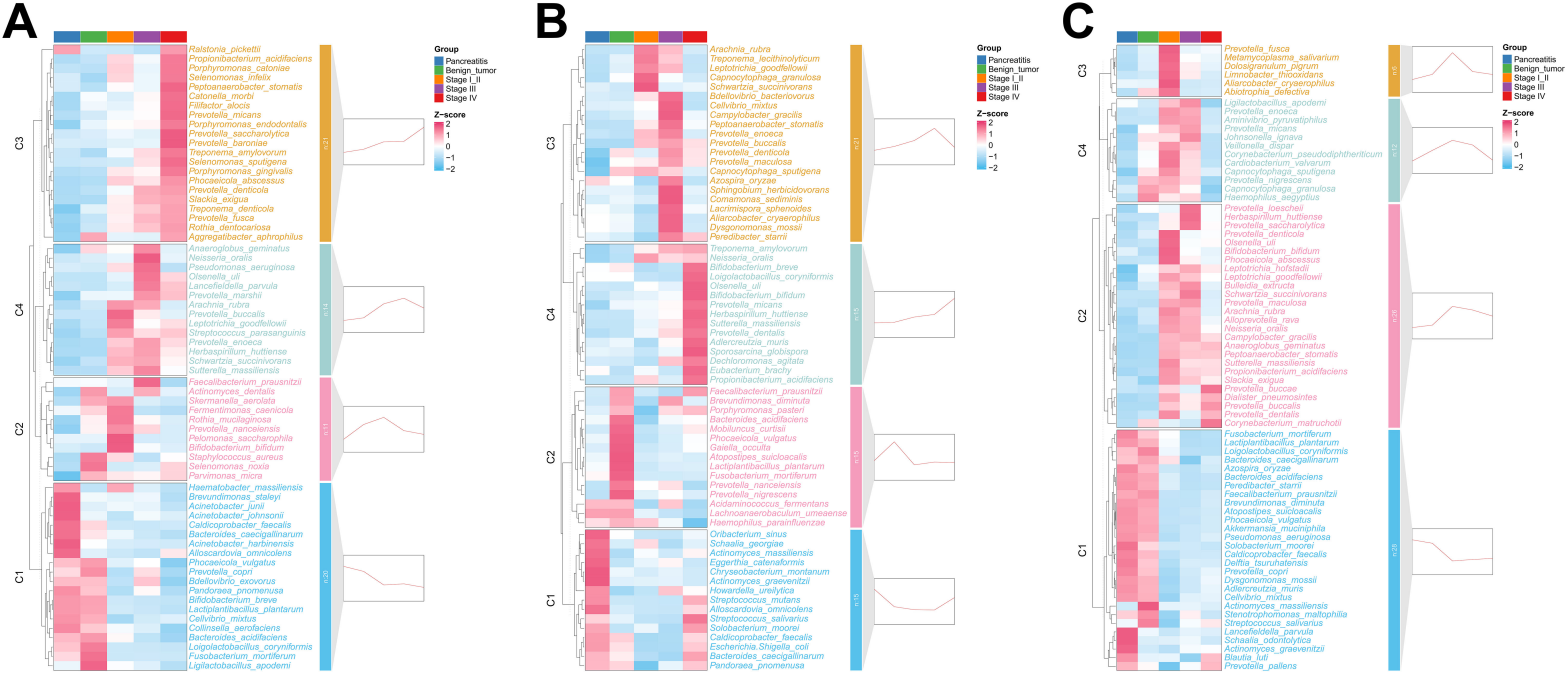
Differential species across the five groups in pancreatic tissue **(A)**, duodenal fluid **(B)**, and saliva **(C)**. The heatmap showed the average relative abundance of differential microbes across the groups, with the z-score representing the row-scaled average relative abundance. The small graphs on the right of each panel displayed the clustering results of the expression trends of the average relative abundance of differential microbes, representing the fitted trend from CP to stage IV PDAC patients for each cluster.

Next, those differently distributed microbial species were clustered into four clusters by the similarity of their abundant-variation pattern and named C1–C4. In the pancreatic tissue, microbes in the C1 including *Haematobacter massiliensis*, *Brevundimonas staleyi*, and *Acinetobacter junii*, maintained a high abundance in CP patients and showed a continuous decrease from CP to stage IV PDAC patients (**Figure 2A** and **Supplementary Table S2**). Microbes in C2 preferred to harbor in stage I/II PDAC patients. Microbes in C3 and C4, which involve a group of well-known periodontitis pathogens such as *Porphyromonas gingivalis*, *Treponema denticola*, *Porphyromonas endodontalis*, and *Filifactor alocis* presented an increasing trend from CP to stage IV PDAC patients. In the duodenal fluid, microbes in the C1 including *Schaalia georgiae* and *Actinomyces massiliensis*, maintained a high abundance in CP patients and showed a continuous decrease from CP to stage III PDAC patients (**Figure 2B** and **Supplementary Table S3**). However, microbes in the C4 including *Treponema amylovorum* and *Neisseria oralis* showed a continuous increase from CP to stage IV PDAC patients. In the saliva, microbes in C1 including *Fusobacterium mortiferum*, *Lactiplantibacillus plantarum*, and *Loigolactobacillus coryniformis* presented a decreasing trend from CP to stage IV PDAC patients (**Figure 2C** and **Supplementary Table S4**). Microbes in C2, C3, and C4 including *Prevotella fusca*, *Metamycoplasma salivarium*, and *Dolosigranulum pigrum* maintained a high abundance in stage I/II PDAC patients. *Peptoanaerobacter stomatis* and *Propionibacterium acidifaciens* showed sequentially increasing trends along PDAC tumorigenesis all in the pancreatic tissue, duodenal fluid, and saliva.

### Variations in the relationship of differential microbes along the development of PDAC

Spearman correlation analysis showed that most of the differential microbes in each cluster had a positive correlation with each other (**Supplementary Figure S3**). In the pancreatic tissue, the microbes in C1 and C3 had more positive correlations than C4. The microbes in C2 had no correlation. In the duodenal fluid, the microbes in C2 and C3 had more positive correlations than C1 and C4. Microbes in C1 and C2 were more positively correlated than C3 and C4 in the saliva.

**Figure 3:**
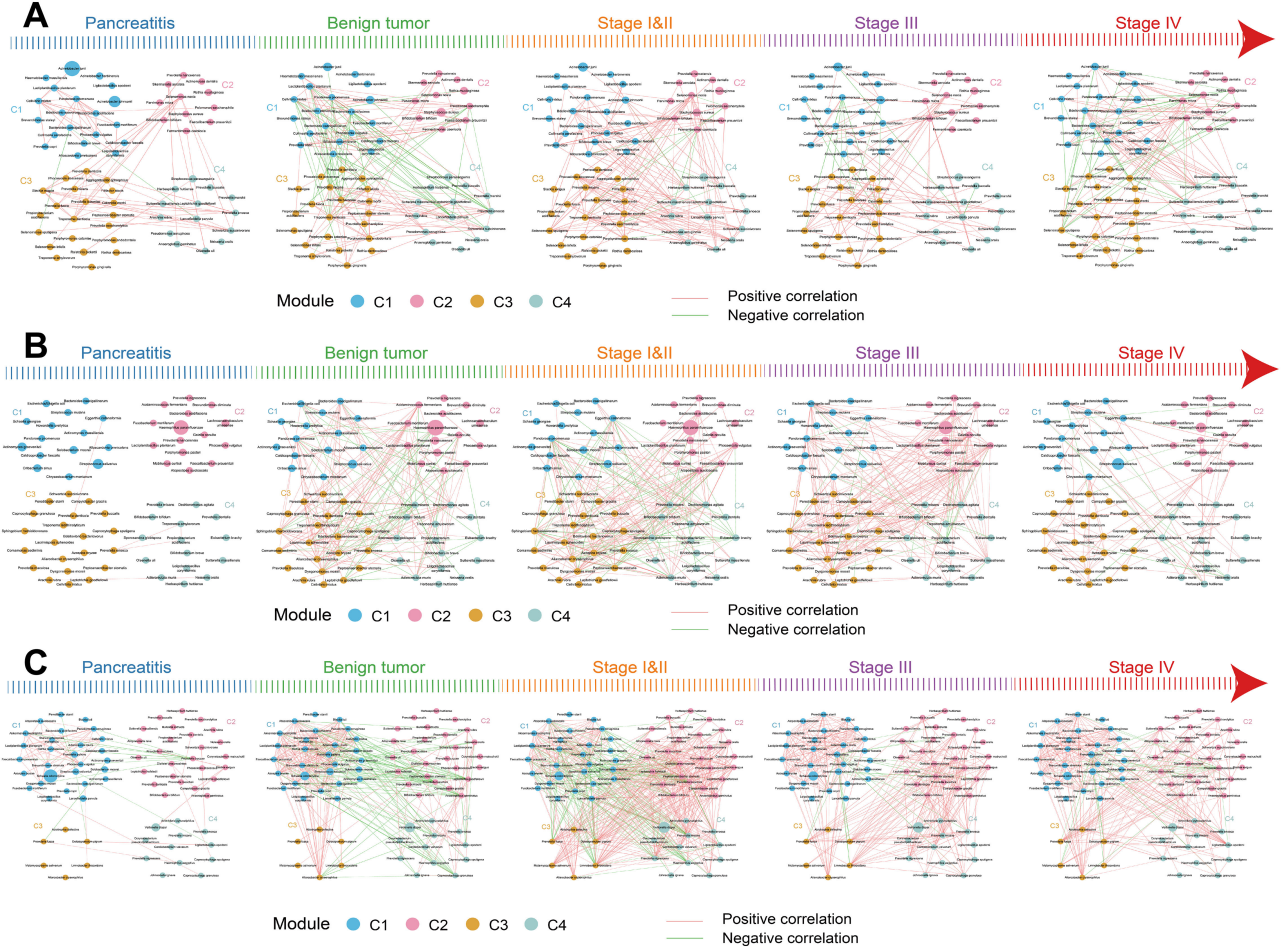
The relationships of differential species in the four clusters across the five groups in the multibody sites. **(A–C)** The interrelationships of differential species between the four clusters in pancreatic tissue **(A)**, duodenal fluid **(B)**, and saliva **(C)** across the five groups. The node sizes represent the mean relative abundance. Lines between nodes represent correlations between the nodes connected by the lines, with red representing positive correlation and green representing negative correlation.

The interrelationships between the four clusters (C1–C4) varied along the development of PDAC. In the pancreatic tissue, most microbes in the four clusters were positively correlated with each other (**Figure 3A**). From CP to stage IV PDAC patients, interrelationships among the four clusters gradually increased and then reduced. Stage I/II PDAC patients had the maximal interrelationships and CP patients had the minimal interrelationships. Microbes in C1 were positively correlated with microbes from C2 but negatively correlated with microbes from C3 and C4 in the benign tumor patients. In the duodenal fluid and saliva, the interrelationships among the four clusters also gradually increased and then reduced along the development of PDAC (**Figures 3B, C**). These results suggest that the relationship among microbes also changed continuously in addition to the changes in the abundance of individual microorganisms with the development of PDAC.

### Changes in ecological communities in the multibody sites along the development of PDAC

**Figure 4:**
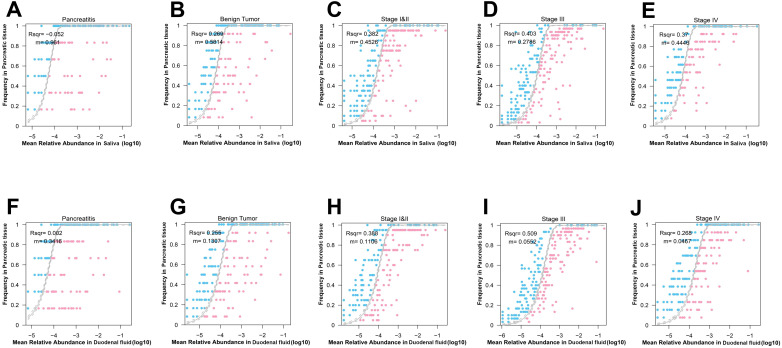
Interactions of the microbes across the five groups in the multibody sites. **(A–E)** Neutral community model (NCM) of salivary microbes to the pancreatic microbial assembly in patients with CP **(A)**, benign tumor **(B)**, stage I/II PDAC **(C)**, stage III PDAC **(D)**, stage IV PDAC **(E)**. The coefficient of determination (*R^2^*) was the goodness of fit of the neutral model. It ranged from ≤ 0 (no fit) to 1 (perfect fit). **(F–J)** NCM of salivary microbes to the duodenal microbial assembly in patients with CP **(F)**, benign tumor **(G)**, stage I/II PDAC **(H)**, stage III PDAC **(I)**, stage IV PDAC **(J)**.

The neutral community model (NCM) was used to measure the relative contribution of the salivary microbes to the pancreatic microbes assembly. The goodness-of-fit (*R^2^*) value of NCM for CP patients was -0.052, that for benign tumor patients was 0.269, that for stage I/II PDAC patients was 0.382, that for stage III PDAC patients was 0.403, and that for stage IV PDAC patients was 0.370 (**Figures 4A–E**). An increase in *R^2^* along the development of PDAC indicates an increase in the relative contribution of the salivary microbes to the assembly of the pancreatic microbes. We also found an increase in the relative contribution of duodenal microbes to the pancreatic microbial assembly from CP, to benign tumors, to stage I/II PDAC, to stage III PDAC patients (*R^2^* increased from 0.002, to 0.255, to 0.388, to 0.509, **Figures 4F–J**).

### Microbial differences in different chemotherapy responses of PDAC patients

**Figure 5:**
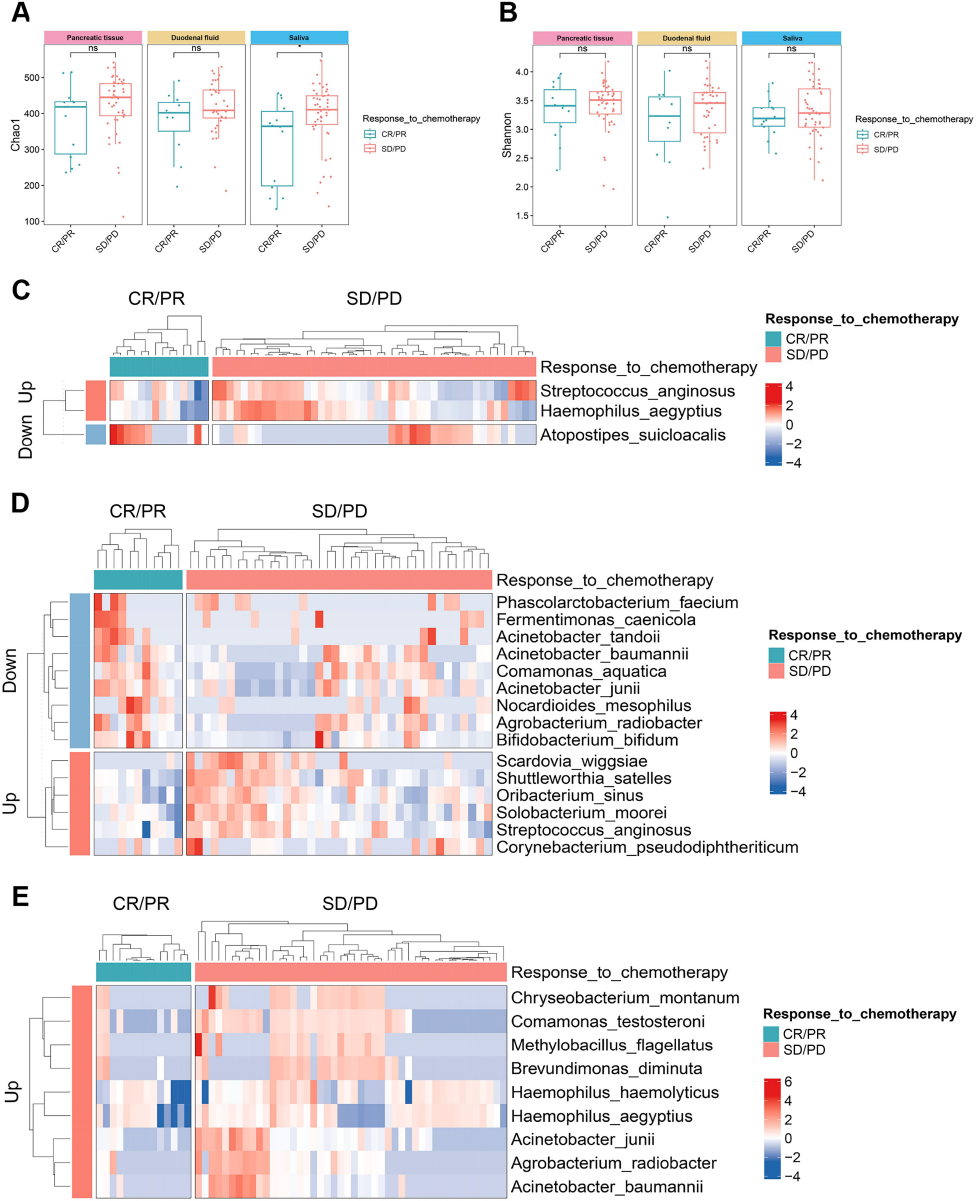
The microbial alpha-diversity and differential species between the groups with different chemotherapy responses in the multibody sites. **(A, B)** The Chao1 **(A)** and Shannon **(B)** indices of alpha-diversity between CR/PR group and SD/PD group in the multibody sites. **(C–E)** Differential species between CR/PR group and SD/PD group in pancreatic tissue **(C)**, duodenal fluid **(D)**, and saliva **(E)**. CR/PR, complete response/partial response; SD/PD, stable disease/progressive disease.

Among the 63 PDAC patients, 60 received standard chemotherapy, of whom 14 were in the CR/PR group and 46 were in the SD/PD group. No significant statistical difference was observed in baseline characteristics between the two groups (**Supplementary Table S5**). We first measured the microbial alpha-diversity among the three sites between the two groups and found the Chao1 index in the saliva of SD/PD patients was significantly higher than that of CR/PR patients (**Figures 5A, B**).

MaAsLin2 analysis detected the differential microbes between the two groups. At the species level, *Streptococcus anginosus* and *Haemophilus aegyptius* were significantly enriched in the pancreatic tissue of the SD/PD patients compared with the CR/PR patients, while *Atopostipes suicloacalis* was significantly reduced (**Figure 5C**). In the duodenal fluid, *Scardovia wiggsiae*, *Shuttleworthia satelles*, and *Oribacterium sinus* increased in the SD/PD patients, whereas *Acinetobacter junii*, *Fermentimonas caenicola*, and *Phascolarctobacterium faecium* decreased (**Figure 5D**). In the saliva, *Chryseobacterium montanum*, *Comamonas testosteroni*, and *Methylobacillus flagellates* were enriched in the SD/PD patients compared with the CR/PR patients (**Figure 5E**). Similar differences were also found across the five stages of PDAC tumorigenesis. *Acinetobacter junii* also presented a decreasing trend from CP to stage IV PDAC patients in the pancreatic tissue. *Fermentimonas caenicola* was also reduced in the pancreatic tissue of stage IV PDAC patients compared with CP patients.

### Microbial differences in PDAC patients with different survival

**Figure 6:**
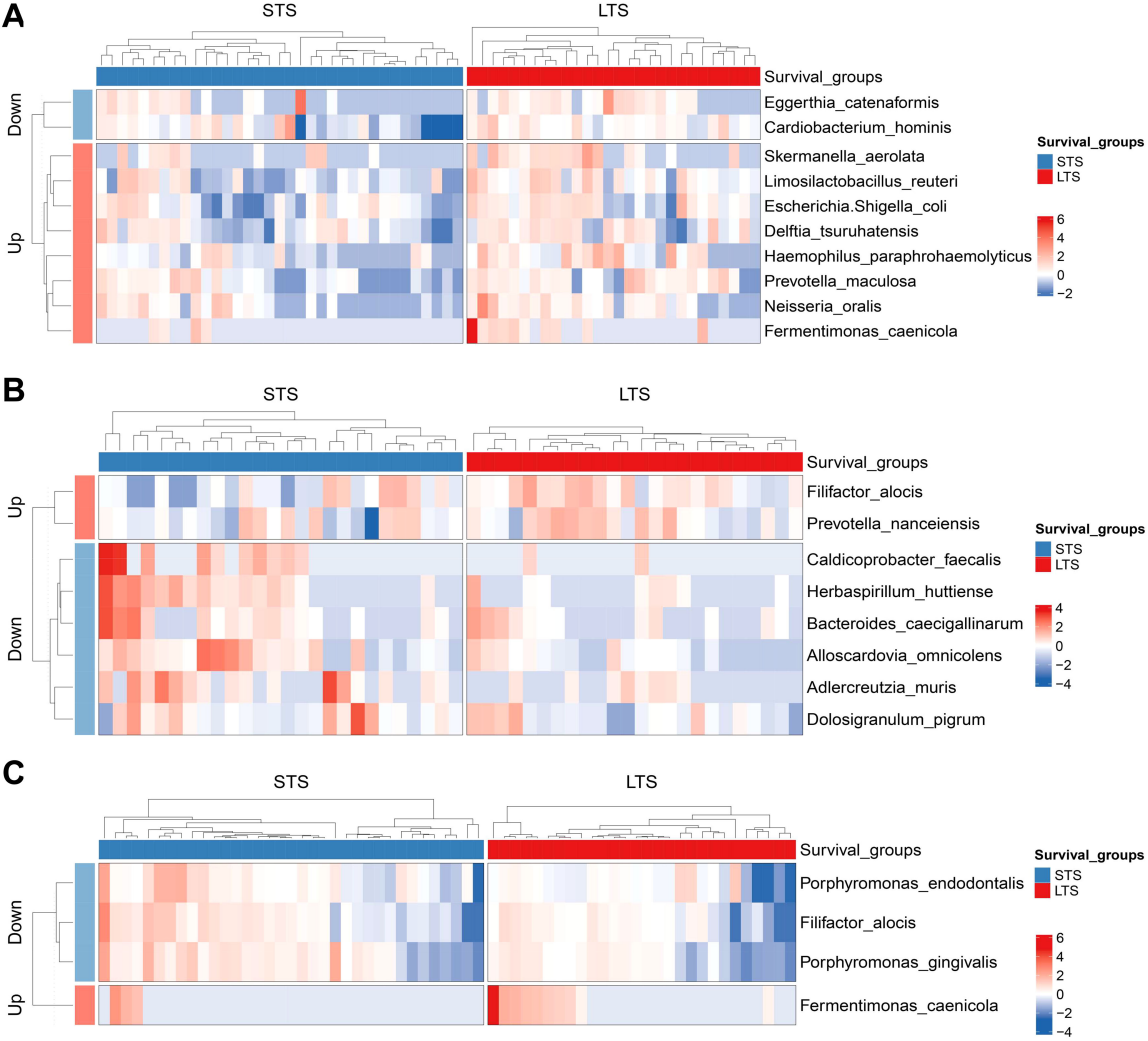
Differential species between the groups with different survival in pancreatic tissue **(A)**, duodenal fluid **(B)**, and saliva **(C)**. LTS, long-term survival; STS, short-term survival.

Among the 63 PDAC patients, 35 were in the STS group and 28 were in the LTS group. No significant statistical difference was observed in baseline characteristics between the two groups (**Supplementary Table S6**). We also measured the microbial alpha-diversity in the pancreatic tissue, duodenal fluid, and saliva between the two groups using different methodologies and found there was no significant difference (**Supplementary Figure S4**). At the species level, MaAsLin2 analysis detected that *Eggerthia catenaformis* and *Cardiobacterium hominis* were significantly enriched, while *Fermentimonas caenicola*, *Skermanella aerolata*, *Limosilactobacillus reuter*, etc. were significantly reduced in the pancreatic tissue of the STS patients compared with the LTS patients (**Figure 6A**). In the duodenal fluid, *Caldicoprobacter faecalis*, *Herbaspirillum huttiense*, etc. increased in the STS patients, whereas *Filifactor alocis* and *Prevotella nanceiensis* decreased (**Figure 6B**). In the saliva, *Porphyromonas gingivalis*, *Porphyromonas endodontalis*, and *Filifactor alocis* were enriched in the STS patients compared with the LTS patients, while *Fermentimonas caenicola* was reduced (**Figure 6C**). Similar differences were also found across the five stages of PDAC tumorigenesis. *Porphyromonas gingivalis*, *Porphyromonas endodontalis*, and *Filifactor alocis* also presented an increasing trend from CP to stage IV PDAC patients in the pancreatic tissue. *Herbaspirillum huttiense* also increased in the duodenual fluid across the five stages of PDAC tumorigenesis. *Fermentimonas caenicola* was also reduced in the pancreatic tissue of stage IV PDAC patients compared with CP patients. *Prevotella nanceiensis* also decreased in the duodenual fluid of stage IV PDAC patients compared with CP patients.

## Discussion

The pathogenesis and early detection of PDAC remain formidable challenges central to ongoing efforts to alleviate the burden of this disease. To our knowledge, this is the first time series analysis of microbiota in pancreatic tissue, duodenal fluid, and saliva across the five stages of PDAC tumorigenesis using pancreatic tissue obtained via EUS-FNA. Our key findings highlighted the significant role of salivary and duodenal microbes in shaping the pancreatic microbial composition associated with PDAC. Notably, we observed continuous increase or decrease of specific species along PDAC tumorigenesis. Furthermore, we found *Porphyromonas gingivalis*, *Porphyromonas endodontalis*, and *Filifactor alocis* presented an increasing trend from CP to stage IV PDAC patients in the pancreatic tissue and were also enriched in the saliva of STS patients. In the duodenal fluid, *Herbaspirillum huttiense* increased sequentially along PDAC tumorigenesis and also increased in the STS patients compared with the LTS patients.

The increased *R^2^* of NCM along PDAC tumorigenesis indicated that salivary and duodenal microbes were significant contributors to the pancreatic microbial composition associated with PDAC. Studies have found microbial translocation active in some diseases, particularly involving the gut microbiota, which can affect various organs through a damaged intestinal barrier (23, 24). In addition, from CP to stage IV PDAC patients, interrelationships among the four clusters gradually increased and then reduced. Previous studies pointed out that communities in which a large proportion of members are connected through positive links tend to be unstable due to positive feedback and co-oscillation in response to environmental fluctuations (25). Therefore, community instability and microbial translocation may happen along PDAC tumorigenesis. However, the precise mechanisms require further elucidation.

Our study confirmed a continuous increase or decrease of specific species along PDAC tumorigenesis. Interestingly, the red complex (*Porphyromonas gingivalis* and *Treponema denticola*), major periodontitis-causing pathogens, presented an increasing trend along PDAC tumorigenesis in the pancreatic tissue (26). And *Porphyromonas gingivalis* was also enriched in the saliva of STS patients compared with the LTS patients. The red complex can secrete peptidyl-arginine deiminase (PAD) to cause the release of neutrophil extracellular traps (NETs). NETs are elevated in both human and murine models of PDAC and associated with poor patient outcomes, metastasis, fibrosis, proliferation, and immune evasion (27, 28). Tan et al. also demonstrated accelerated tumor growth in PDAC murine models when exposed to *Porphyromonas gingivalis* via oral gavage. They confirmed the localization of bacteria to the pancreas and a resultant pro-inflammatory tumor microenvironment (TME) characterized by a neutrophil-dominated milieu (29). Other periodontal pathogens, including *Peptoanaerobacter stomatis*, *Slackia exigua*, *Propionibacterium acidifaciens*, *Catonella morbi*, *Filifactor alocis*, and *Rothia dentocariosa*, related to oral diseases like periodontitis or oral cavity squamous cell carcinoma (OSCC), were also increased sequentially from CP to stage IV PDAC patients in the pancreatic tissue (30–35). The sequentially increasing trends along PDAC tumorigenesis of *Peptoanaerobacter stomatis* and *Propionibacterium acidifaciens* were also observed in the duodenal fluid and saliva. In addition, *Prevotella dentalis* can promote the development of colorectal cancer (CRC) (36), while *Slackia exigua* is associated with gastric cancer (GC) (37). *Porphyromonas endodontalis* was identified as the new CRC driver species and the top microbial biomarker to differentiate esophageal squamous carcinoma (ESC) from controls (38, 39). In contrast, bacteria commonly used as probiotics *Bifidobacterium breve* and *Lactiplantibacillus plantarum* presented a decreasing trend along PDAC tumorigenesis in the pancreatic tissue. Indole-3-lactic acid derived from *Bifidobacterium breve* and *Lactobacillus plantarum* can ameliorate colorectal tumorigenesis via epigenetic regulation of CD8+ T cell immunity (40, 41). Whether these microbes are involved in the occurrence of PDAC needs to be further studied.

Our study had several limitations. First, the sample size was limited. Second, it remains unclear whether the observed microbial differences are the causes or consequences of PDAC. Further *in vivo* and *in vitro* research is needed to substantiate our findings.

To our knowledge, this is the first time series analysis to describe microbiota in pancreatic tissue, duodenal fluid, and saliva across the five stages of PDAC tumorigenesis. Further studies regarding the functions of the above-mentioned microbes were warranted as the results may reveal the mechanisms underlying the occurrence and development of PDAC and provide a basis for future treatments.

## Conclusions

Salivary and duodenal microorganisms play a significant role in shaping the pancreatic microbial environment in the context of PDAC. Notably, *Porphyromonas gingivalis*, *Porphyromonas endodontalis*, and *Filifactor alocis*, which sequentially increased in pancreatic tissue from CP to stage IV PDAC patients, were also enriched in saliva of patients with STS compared with those with LTS. Further investigation into these microbial entities is essential for elucidating their precise roles in PDAC development, which may provide valuable insights for future therapeutic interventions.

## Data Availability

The data presented in the study are deposited in the Figshare repository, DOI number 10.6084/m9.figshare.26798644.
