# Supplementary material for: Succession of the multi-site microbiome along pancreatic ductal adenocarcinoma tumorigenesis
Source: Front Immunol. 2024 Nov 7;15:1487242. doi: 10.3389/fimmu.2024.1487242 (PMC11580624; doi:10.3389/fimmu.2024.1487242)
Supplement: Supplementary file 1 [file DataSheet1.pdf]

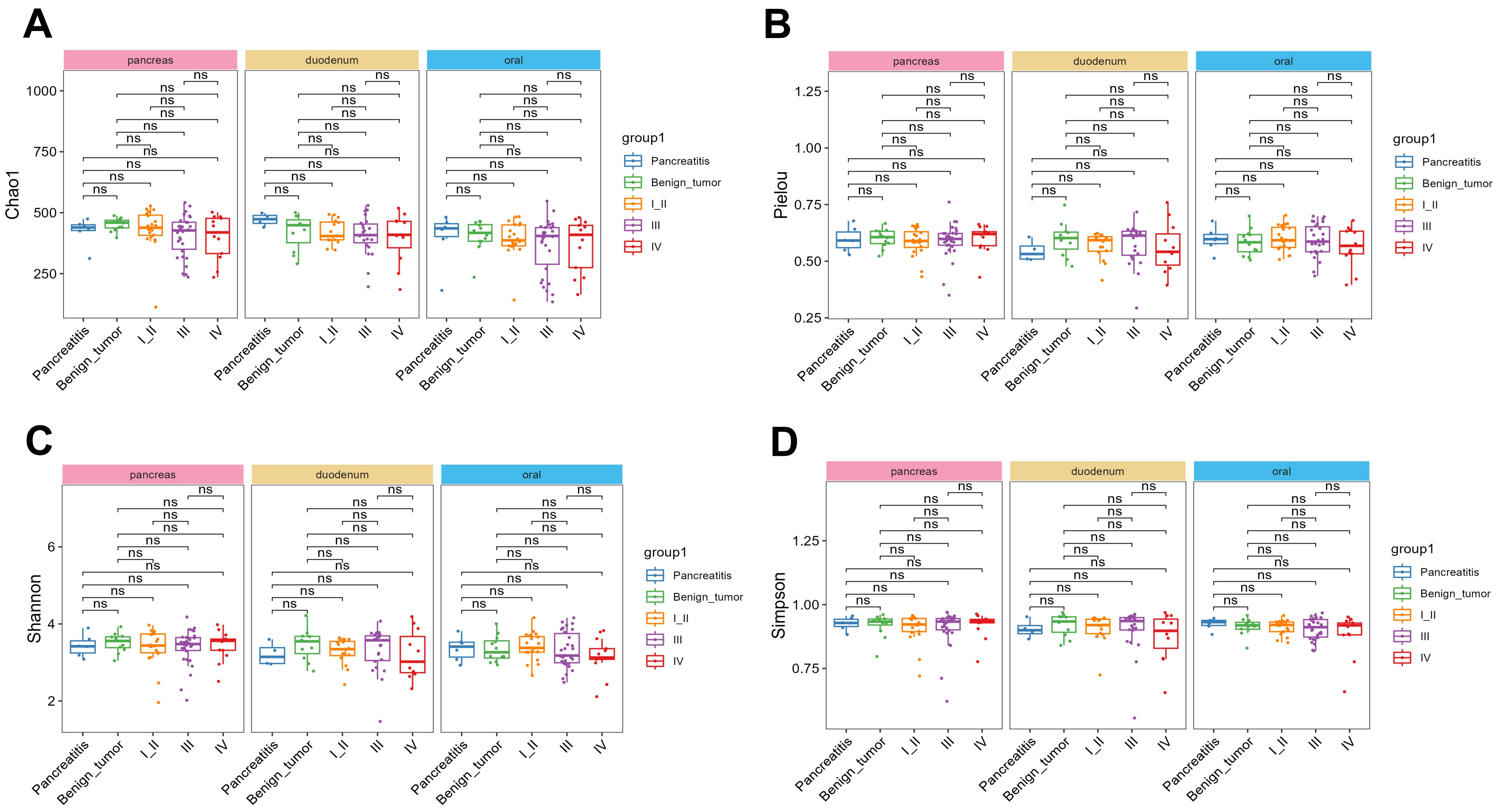

Fig. S1 The microbial alpha-diversity across the five groups in pancreatic tissue, duodenal fluid, and saliva by different metrics, including Chao1 (A), Pielou (B), Shannon (C), and Simpson (D).

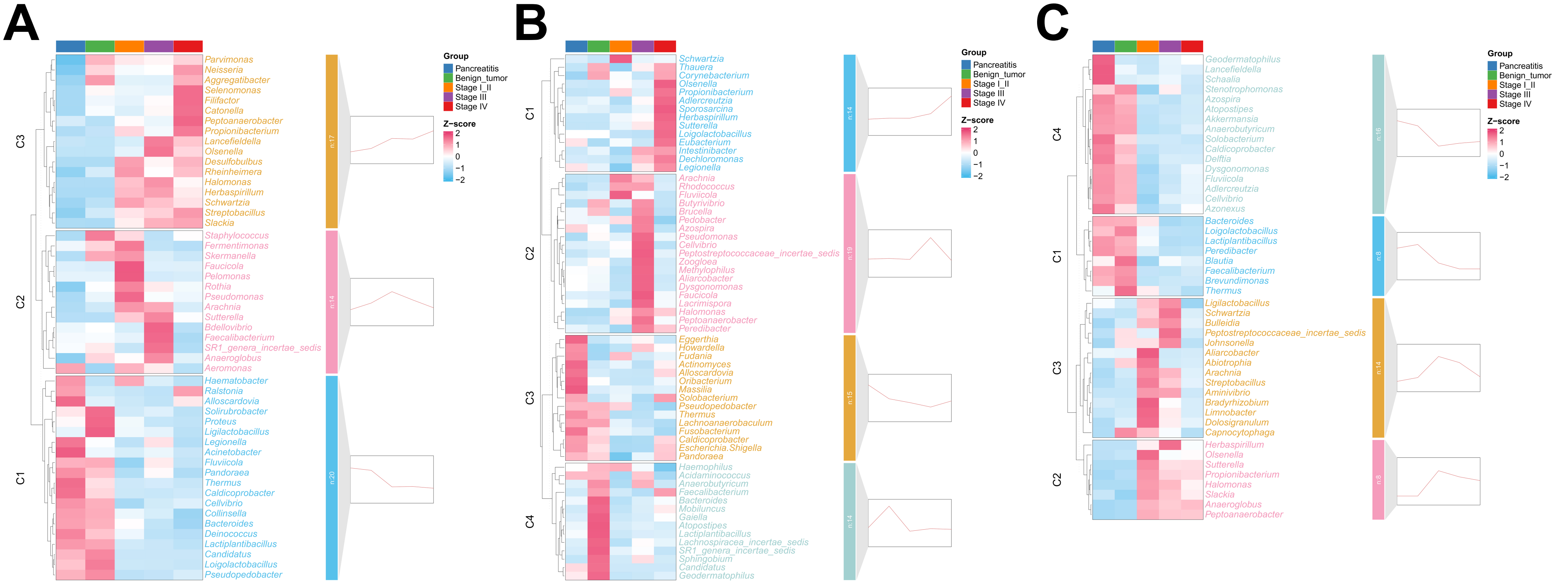

Fig. S2 Differential genera across the five groups in pancreatic tissue (A), duodenal fluid (B), and saliva (C).

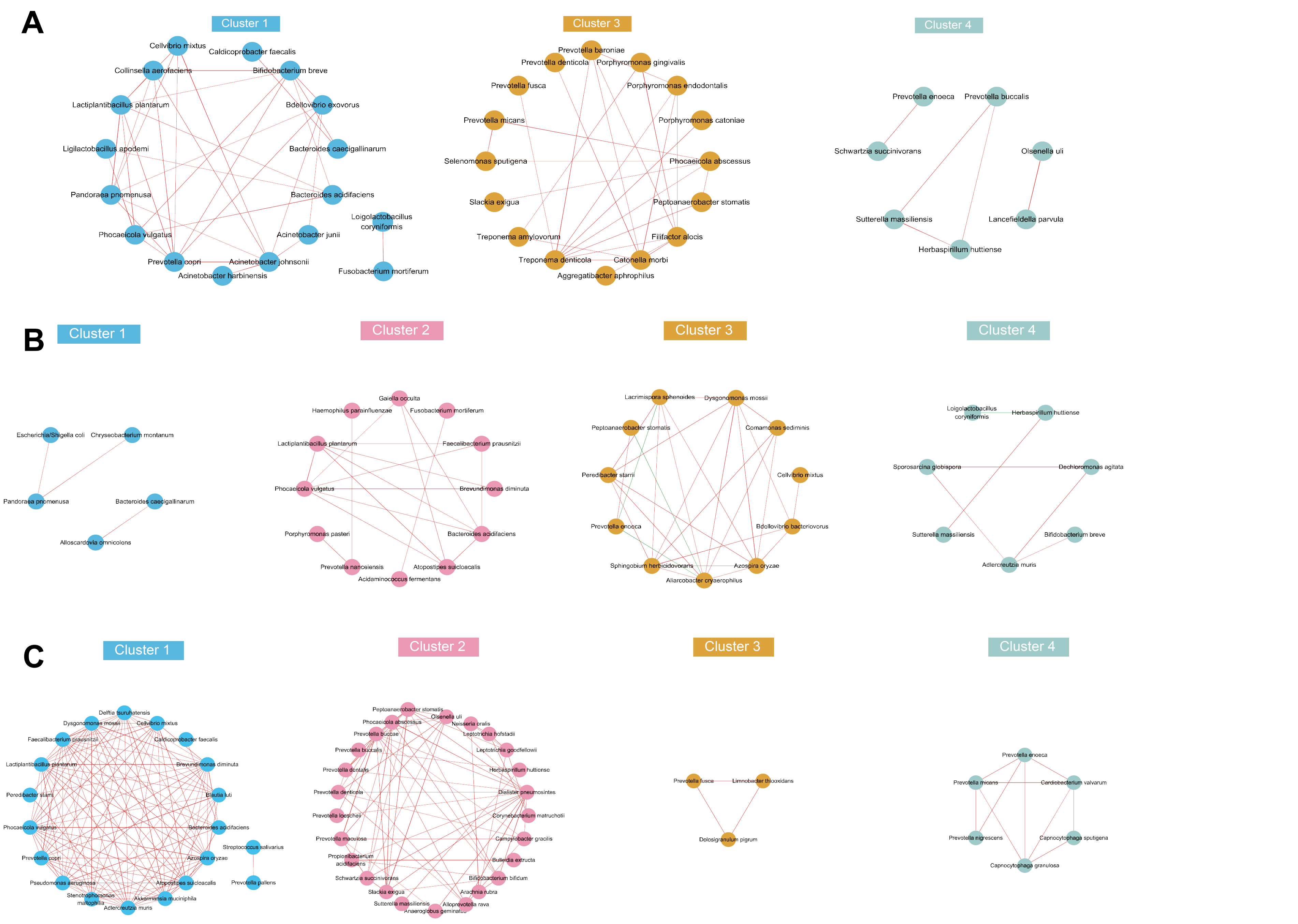

Fig. S3 The relationships of differential species within individual cluster in pancreatic tissue (A), duodenal fluid (B), and saliva (C) across the five groups.

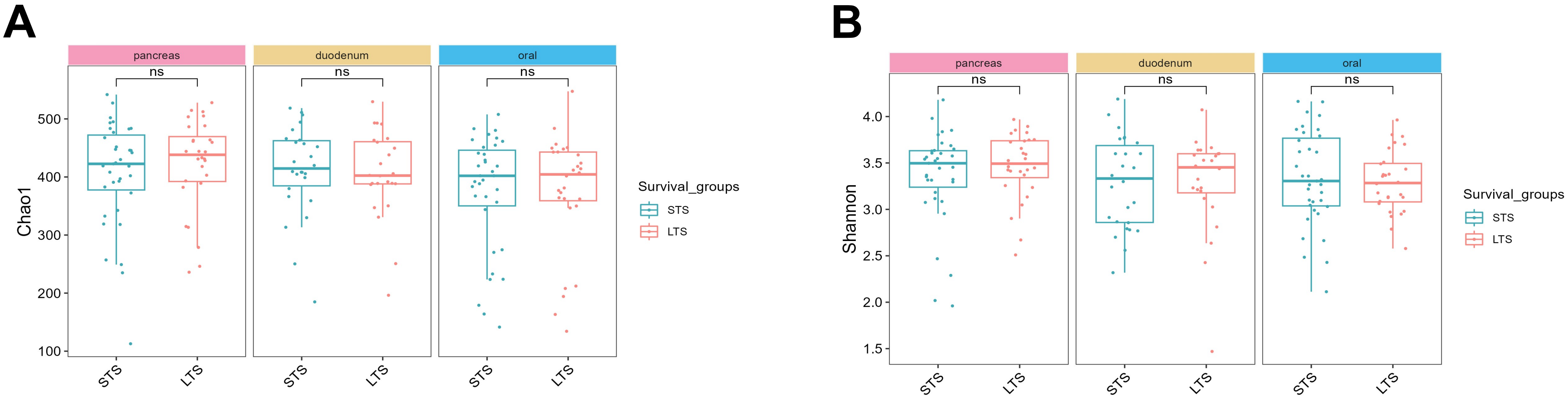

Fig. S4 The Chao1 (A) and Shannon (B) indices of alpha-diversity between STS group and LTS group in the multibody sites.
