# Supplementary material for: Succession of the multi-site microbiome along pancreatic ductal adenocarcinoma tumorigenesis
Source: Front Immunol. 2024 Nov 7;15:1487242. doi: 10.3389/fimmu.2024.1487242 (PMC11580624; doi:10.3389/fimmu.2024.1487242)
Supplement: Supplementary file 2 [file Table1.docx]

**Table S1 Baseline characteristics among the five groups.**

|  | Chronic  pancreatitis (n = 6) | Pancreatic benign tumors (n = 12) | Stage Ⅰ/Ⅱ PDAC (n = 20) | Stage Ⅲ PDAC (n = 30) | Stage Ⅳ PDAC (n = 13) | P |
| --- | --- | --- | --- | --- | --- | --- |
| Age (ys, mean ± SD) | 59.17 ± 3.81 | 54.00 ± 4.24 | 58.75 ± 2.45 | 56.77 ± 1.99 | 65.00 ± 1.74 | 0.118 |
| Gender (n, %) |  |  |  |  |  | 0.834 |
| Male | 5 | 8 | 13 | 20 | 7 |  |
| Female | 1 | 4 | 7 | 10 | 6 |  |
| BMI (mean ± SD) | 21.85 ± 1.70 | 24.12 ± 0.96 | 23.33 ± 0.67 | 23.56 ± 0.55 | 20.49 ± 1.12 | 0.068 |
| CA19-9 (U/ml, mean ± SD) | 497.10 ± 213.98 | 13.44 ± 3.95 | 345.54 ± 91.75 | 344.91 ± 75.69 | 558.93 ± 127.34 | 0.004 |
| CEA (ng/ml, mean ± SD) | 4.72 ± 2.42 | 2.00 ± 0.49 | 5.32 ± 1.44 | 7.43 ± 3.84 | 15.85 ± 6.88 | 0.075 |
| CHO (mmol/L, mean ± SD) | 4.69 ± 0.52 | 4.75 ± 0.59 | 4.99 ± 0.53 | 4.33 ± 0.24 | 5.93 ± 1.17 | 0.560 |
| LDL (mmol/L, mean ± SD) | 2.79 ± 0.38 | 2.85 ± 0.36 | 3.05 ± 0.40 | 2.57 ± 0.21 | 3.97 ± 1.15 | 0.685 |
| Diabetes |  |  |  |  |  | 0.788 |
| Yes | 3 | 3 | 7 | 9 | 3 |  |
| No  Smoking  Yes  No  Drinking  Yes  No | 3  2  4  4  2 | 9  3  9  6  6 | 13  8  12  9  11 | 21  12  18  15  15 | 10  4  9  5  8 | 0.913  0.853 |

PDAC, pancreatic ductal adenocarcinoma; BMI, body mass index; CA 19-9, carbohydrate antigen 19–9; CEA, carcinoembryonic antigen; CHO, total cholesterol; LDL, low-density lipoprotein cholesterol.

**Table S2 Differential genera and species across the five groups in pancreatic tissue.**

|  | **Genus** | | | | | | | | | |
| --- | --- | --- | --- | --- | --- | --- | --- | --- | --- | --- |
| **Microbes** | | **Groups** | **coef** | **stderr** | **p-value** | | **q-value** | | **p-adjust** | |
| Candidatus | | III | -3.428761374 | 0.351556261 | | 1.0512E-14 | | 7.31638E-12 | | 7.31638E-12 |
| Candidatus | | I_II | -3.434776364 | 0.370192475 | | 6.92529E-14 | | 2.41E-11 | | 2.41E-11 |
| Candidatus | | IV | -3.44766325 | 0.389585621 | | 3.25958E-13 | | 7.56223E-11 | | 7.56223E-11 |
| Loigolactobacillus | | III | -3.666080208 | 0.53874612 | | 2.26309E-09 | | 3.15022E-07 | | 3.15022E-07 |
| Loigolactobacillus | | IV | -4.083592352 | 0.598062387 | | 2.04422E-09 | | 3.15022E-07 | | 3.15022E-07 |
| Loigolactobacillus | | I_II | -3.491101127 | 0.564852684 | | 3.16646E-08 | | 3.67309E-06 | | 3.67309E-06 |
| Pseudopedobacter | | IV | -2.530461188 | 0.677981594 | | 0.000380514 | | 0.037833958 | | 0.037833958 |
| Caldicoprobacter | | III | -2.134375518 | 0.581986569 | | 0.000481566 | | 0.041896281 | | 0.041896281 |
| Pseudopedobacter | | III | -2.154357192 | 0.604107795 | | 0.000681879 | | 0.052731944 | | 0.052731944 |
| Caldicoprobacter | | I_II | -2.125341065 | 0.616190458 | | 0.000940529 | | 0.065460842 | | 0.065460842 |
| Bacteroides | | IV | -3.973656364 | 1.17731465 | | 0.001331533 | | 0.084249746 | | 0.084249746 |
| Thermus | | III | -1.781707766 | 0.542339946 | | 0.00206424 | | 0.119725944 | | 0.119725944 |
| Pseudopedobacter | | I_II | -2.01835823 | 0.645309104 | | 0.002564733 | | 0.137311858 | | 0.137311858 |
| Acinetobacter | | IV | -3.442334532 | 1.15115242 | | 0.004139611 | | 0.171498003 | | 0.171498003 |
| Aeromonas | | IV | -3.588134859 | 1.269512631 | | 0.00714575 | | 0.171498003 | | 0.171498003 |
| Aggregatibacter | | IV | 4.214633797 | 1.450850417 | | 0.004827961 | | 0.171498003 | | 0.171498003 |
| Arachnia | | I_II | 2.120505463 | 0.761216487 | | 0.006761543 | | 0.171498003 | | 0.171498003 |
| Bacteroides | | III | -3.035087122 | 0.994346654 | | 0.004466556 | | 0.171498003 | | 0.171498003 |
| Catonella | | IV | 3.619089696 | 1.272581966 | | 0.006193189 | | 0.171498003 | | 0.171498003 |
| Collinsella | | III | -2.822653544 | 1.017791465 | | 0.007014371 | | 0.171498003 | | 0.171498003 |
| Deinococcus | | III | -2.159099981 | 0.72772472 | | 0.004234079 | | 0.171498003 | | 0.171498003 |
| Deinococcus | | IV | -2.41517553 | 0.825559307 | | 0.004566251 | | 0.171498003 | | 0.171498003 |
| Herbaspirillum | | IV | 2.036484646 | 0.708424873 | | 0.005450982 | | 0.171498003 | | 0.171498003 |
| Lactiplantibacillus | | IV | -3.894553149 | 1.346057436 | | 0.004996011 | | 0.171498003 | | 0.171498003 |
| Neisseria | | Benign_tumor | 3.190344396 | 1.105766594 | | 0.006778427 | | 0.171498003 | | 0.171498003 |
| Olsenella | | IV | 2.932227024 | 1.059265255 | | 0.007098627 | | 0.171498003 | | 0.171498003 |
| Rothia | | I_II | 1.770395479 | 0.624954297 | | 0.005924286 | | 0.171498003 | | 0.171498003 |
| Thermus | | I_II | -1.68186781 | 0.597197094 | | 0.007109841 | | 0.171498003 | | 0.171498003 |
| Thermus | | IV | -1.89926223 | 0.633078618 | | 0.004222706 | | 0.171498003 | | 0.171498003 |
| Halomonas | | III | 1.630018727 | 0.591757408 | | 0.007531985 | | 0.174742046 | | 0.174742046 |
| Aeromonas | | Benign_tumor | -3.239809308 | 1.214587861 | | 0.012505111 | | 0.189102768 | | 0.189102768 |
| Aeromonas | | III | -2.854615031 | 1.027937242 | | 0.011862973 | | 0.189102768 | | 0.189102768 |
| Aggregatibacter | | Benign_tumor | 3.760357626 | 1.469572279 | | 0.012526833 | | 0.189102768 | | 0.189102768 |
| Anaeroglobus | | I_II | 2.274447494 | 0.867359294 | | 0.01058392 | | 0.189102768 | | 0.189102768 |
| Anaeroglobus | | III | 2.117238216 | 0.821266894 | | 0.011921186 | | 0.189102768 | | 0.189102768 |
| Caldicoprobacter | | IV | -1.641144067 | 0.65100731 | | 0.013856668 | | 0.189102768 | | 0.189102768 |
| Collinsella | | IV | -2.99285747 | 1.1290218 | | 0.009805062 | | 0.189102768 | | 0.189102768 |
| Filifactor | | IV | 3.794451163 | 1.407965316 | | 0.008796293 | | 0.189102768 | | 0.189102768 |
| Fluviicola | | I_II | -3.569057023 | 1.369093008 | | 0.011018437 | | 0.189102768 | | 0.189102768 |
| Haematobacter | | IV | -2.679590751 | 0.985616209 | | 0.00880231 | | 0.189102768 | | 0.189102768 |
| Halomonas | | IV | 1.744845056 | 0.662431739 | | 0.010330992 | | 0.189102768 | | 0.189102768 |
| Lactiplantibacillus | | III | -3.156137406 | 1.213839566 | | 0.011238922 | | 0.189102768 | | 0.189102768 |
| Legionella | | I_II | -2.677921739 | 1.056109084 | | 0.013393178 | | 0.189102768 | | 0.189102768 |
| Pandoraea | | I_II | -2.372563614 | 0.924999466 | | 0.012383985 | | 0.189102768 | | 0.189102768 |
| Propionibacterium | | IV | 1.967131471 | 0.779708585 | | 0.013785319 | | 0.189102768 | | 0.189102768 |
| Proteus | | IV | -1.625536438 | 0.620825119 | | 0.010915365 | | 0.189102768 | | 0.189102768 |
| Pseudomonas | | I_II | 2.737663042 | 1.079535597 | | 0.013301396 | | 0.189102768 | | 0.189102768 |
| Selenomonas | | IV | 2.314649327 | 0.870668845 | | 0.009628847 | | 0.189102768 | | 0.189102768 |
| Staphylococcus | | Benign_tumor | 4.731633288 | 1.783539741 | | 0.00988184 | | 0.189102768 | | 0.189102768 |
| Sutterella | | I_II | 1.797892399 | 0.710308455 | | 0.013498326 | | 0.189102768 | | 0.189102768 |
| Sutterella | | III | 1.743190123 | 0.674035176 | | 0.01167621 | | 0.189102768 | | 0.189102768 |
| Alloscardovia | | III | -2.16145859 | 0.861891103 | | 0.014706818 | | 0.189554538 | | 0.189554538 |
| Proteus | | III | -1.389525869 | 0.548876493 | | 0.014400199 | | 0.189554538 | | 0.189554538 |
| Ralstonia | | III | -2.834954942 | 1.11147836 | | 0.014692582 | | 0.189554538 | | 0.189554538 |
| Haematobacter | | Benign_tumor | -2.457115773 | 0.969672206 | | 0.015448121 | | 0.195488953 | | 0.195488953 |
| Skermanella | | I_II | 2.857490461 | 1.159030827 | | 0.016011886 | | 0.199004865 | | 0.199004865 |
| Ligilactobacillus | | IV | -3.469364343 | 1.415327937 | | 0.016569205 | | 0.202318714 | | 0.202318714 |
| Sutterella | | IV | 1.828525582 | 0.748877753 | | 0.017000802 | | 0.204009627 | | 0.204009627 |
| Streptobacillus | | I_II | 1.8488008 | 0.759019091 | | 0.017883957 | | 0.210970065 | | 0.210970065 |
| Cellvibrio | | I_II | -1.920952118 | 0.800523174 | | 0.01895693 | | 0.21877002 | | 0.21877002 |
| Neisseria | | I_II | 2.606576764 | 1.032498725 | | 0.019173809 | | 0.21877002 | | 0.21877002 |
| Acinetobacter | | III | -2.391828732 | 0.979112288 | | 0.020008806 | | 0.221049664 | | 0.221049664 |
| Halomonas | | I_II | 1.502106676 | 0.630310937 | | 0.019812693 | | 0.221049664 | | 0.221049664 |
| Herbaspirillum | | I_II | 1.591531274 | 0.672398837 | | 0.021003992 | | 0.228418416 | | 0.228418416 |
| Acinetobacter | | I_II | -2.563689118 | 1.088452649 | | 0.021818659 | | 0.231025078 | | 0.231025078 |
| Pelomonas | | I_II | -2.731452889 | 1.165707884 | | 0.02190755 | | 0.231025078 | | 0.231025078 |
| Lactiplantibacillus | | I_II | -2.971146779 | 1.272368555 | | 0.022239928 | | 0.231029703 | | 0.231029703 |
| Aggregatibacter | | I_II | 3.180704453 | 1.376449894 | | 0.023596444 | | 0.232979779 | | 0.232979779 |
| Fermentimonas | | IV | -1.691835721 | 0.728004321 | | 0.022853919 | | 0.232979779 | | 0.232979779 |
| Peptoanaerobacter | | I_II | 2.002958251 | 0.860049869 | | 0.023560513 | | 0.232979779 | | 0.232979779 |
| Slackia | | I_II | 1.956205151 | 0.847622742 | | 0.023766615 | | 0.232979779 | | 0.232979779 |
| Arachnia | | III | 1.657670203 | 0.726902971 | | 0.025430143 | | 0.245824717 | | 0.245824717 |
| Collinsella | | I_II | -2.353853466 | 1.066942316 | | 0.030466935 | | 0.255548091 | | 0.255548091 |
| Faecalibacterium | | IV | -3.003780062 | 1.368934443 | | 0.031314416 | | 0.255548091 | | 0.255548091 |
| Faucicola | | I_II | 3.339056669 | 1.473412395 | | 0.029784162 | | 0.255548091 | | 0.255548091 |
| Haematobacter | | III | -1.946179315 | 0.838718147 | | 0.027653459 | | 0.255548091 | | 0.255548091 |
| Herbaspirillum | | III | 1.404462019 | 0.623890097 | | 0.028145331 | | 0.255548091 | | 0.255548091 |
| Lancefieldella | | III | 2.546894424 | 1.140497201 | | 0.029797719 | | 0.255548091 | | 0.255548091 |
| Legionella | | III | -2.201432532 | 0.991174474 | | 0.030034285 | | 0.255548091 | | 0.255548091 |
| Neisseria | | III | 2.22372696 | 0.907930373 | | 0.029147424 | | 0.255548091 | | 0.255548091 |
| Neisseria | | IV | 2.525750479 | 1.061808229 | | 0.030913652 | | 0.255548091 | | 0.255548091 |
| Olsenella | | III | 2.142538143 | 0.947710337 | | 0.027128467 | | 0.255548091 | | 0.255548091 |
| Propionibacterium | | I_II | 1.643825986 | 0.741159625 | | 0.029625908 | | 0.255548091 | | 0.255548091 |
| Rheinheimera | | I_II | 2.486964145 | 1.135208203 | | 0.031576345 | | 0.255548091 | | 0.255548091 |
| Rothia | | III | 1.322664536 | 0.596720364 | | 0.029695011 | | 0.255548091 | | 0.255548091 |
| Solirubrobacter | | I_II | -2.102368509 | 0.956041306 | | 0.031104425 | | 0.255548091 | | 0.255548091 |
| Filifactor | | III | 2.736961329 | 1.254135674 | | 0.032745496 | | 0.259581409 | | 0.259581409 |
| SR1_genera_incertae_sedis | | IV | -2.376953155 | 1.092854453 | | 0.032820638 | | 0.259581409 | | 0.259581409 |
| Legionella | | IV | -2.414227593 | 1.117552648 | | 0.033981836 | | 0.265745591 | | 0.265745591 |
| Selenomonas | | I_II | 1.773100092 | 0.82335262 | | 0.034585807 | | 0.267463571 | | 0.267463571 |
| Filifactor | | I_II | 2.874259801 | 1.340821233 | | 0.035555093 | | 0.268982008 | | 0.268982008 |
| Fluviicola | | III | -2.786737805 | 1.298423523 | | 0.035481958 | | 0.268982008 | | 0.268982008 |
| Bdellovibrio | | I_II | -2.200429347 | 1.033438414 | | 0.037680858 | | 0.280648197 | | 0.280648197 |
| Schwartzia | | I_II | 2.122882778 | 1.004429213 | | 0.037903636 | | 0.280648197 | | 0.280648197 |
| Olsenella | | I_II | 2.079617537 | 1.003373839 | | 0.041658009 | | 0.305199732 | | 0.305199732 |
| Rothia | | IV | 1.350681927 | 0.659074197 | | 0.043928394 | | 0.318480859 | | 0.318480859 |
| Anaeroglobus | | IV | 1.841343254 | 0.910626558 | | 0.046737411 | | 0.327658605 | | 0.327658605 |
| Desulfobulbus | | I_II | 1.146719124 | 0.566544439 | | 0.047674729 | | 0.327658605 | | 0.327658605 |
| Faecalibacterium | | III | -2.518956054 | 1.240418256 | | 0.045827288 | | 0.327658605 | | 0.327658605 |
| Parvimonas | | IV | 1.765846983 | 0.860753653 | | 0.047438471 | | 0.327658605 | | 0.327658605 |
| Staphylococcus | | I_II | 3.378890981 | 1.680218603 | | 0.048018934 | | 0.327658605 | | 0.327658605 |
| Streptobacillus | | IV | 1.630075565 | 0.8054077 | | 0.047342685 | | 0.327658605 | | 0.327658605 |
|  | **Species** | | | | | | | | | |
| Loigolactobacillus_coryniformis | | III | -3.666080208 | 0.53874612 | 2.26309E-09 | | 9.41446E-07 | | 9.41446E-07 | |
| Loigolactobacillus_coryniformis | | IV | -4.083592352 | 0.598062387 | 2.04422E-09 | | 9.41446E-07 | | 9.41446E-07 | |
| Loigolactobacillus_coryniformis | | I_II | -3.491101127 | 0.564852684 | 3.16646E-08 | | 8.78165E-06 | | 8.78165E-06 | |
| Fusobacterium_mortiferum | | III | -2.419742935 | 0.445343291 | 2.37422E-06 | | 0.00039507 | | 0.00039507 | |
| Fusobacterium_mortiferum | | IV | -2.71586076 | 0.510263759 | 1.97808E-06 | | 0.00039507 | | 0.00039507 | |
| Fusobacterium_mortiferum | | I_II | -2.457656375 | 0.484172323 | 5.78504E-06 | | 0.000802192 | | 0.000802192 | |
| Prevotella_buccalis | | I_II | 2.527763531 | 0.673780619 | 0.000347198 | | 0.041267016 | | 0.041267016 | |
| Caldicoprobacter_faecalis | | III | -2.134375518 | 0.581986569 | 0.000481566 | | 0.050082911 | | 0.050082911 | |
| Caldicoprobacter_faecalis | | I_II | -2.125341065 | 0.616190458 | 0.000940529 | | 0.086946713 | | 0.086946713 | |
| Bifidobacterium_breve | | III | -2.649346038 | 0.80450336 | 0.001687777 | | 0.140423018 | | 0.140423018 | |
| Actinomyces_dentalis | | I_II | 2.492874549 | 0.630511837 | 0.002333183 | | 0.161767361 | | 0.161767361 | |
| Prevotella_buccalis | | III | 2.023588962 | 0.636353208 | 0.002192432 | | 0.161767361 | | 0.161767361 | |
| Acinetobacter_johnsonii | | I_II | -3.801345618 | 1.387594531 | 0.007697458 | | 0.188361337 | | 0.188361337 | |
| Aggregatibacter_aphrophilus | | IV | 4.21964318 | 1.461541087 | 0.005076744 | | 0.188361337 | | 0.188361337 | |
| Arachnia_rubra | | I_II | 2.120505463 | 0.761216487 | 0.006761543 | | 0.188361337 | | 0.188361337 | |
| Bacteroides_acidifaciens | | IV | -3.543720605 | 1.225942496 | 0.005288608 | | 0.188361337 | | 0.188361337 | |
| Bdellovibrio_exovorus | | IV | -2.154125374 | 0.746662709 | 0.007117423 | | 0.188361337 | | 0.188361337 | |
| Bifidobacterium_bifidum | | I_II | 2.347770952 | 0.813363707 | 0.005096207 | | 0.188361337 | | 0.188361337 | |
| Bifidobacterium_breve | | IV | -2.753064404 | 0.914382356 | 0.003575644 | | 0.188361337 | | 0.188361337 | |
| Catonella_morbi | | IV | 3.619089696 | 1.272581966 | 0.006193189 | | 0.188361337 | | 0.188361337 | |
| Collinsella_aerofaciens | | III | -2.762948114 | 1.004639408 | 0.007479145 | | 0.188361337 | | 0.188361337 | |
| Herbaspirillum_huttiense | | IV | 2.036484646 | 0.708424873 | 0.005450982 | | 0.188361337 | | 0.188361337 | |
| Lactiplantibacillus_plantarum | | IV | -3.894553149 | 1.346057436 | 0.004996011 | | 0.188361337 | | 0.188361337 | |
| Leptotrichia_goodfellowii | | I_II | 2.225823102 | 0.789506755 | 0.006158712 | | 0.188361337 | | 0.188361337 | |
| Neisseria_oralis | | I_II | 3.146636882 | 1.087866443 | 0.00743883 | | 0.188361337 | | 0.188361337 | |
| Olsenella_uli | | IV | 2.932227024 | 1.059265255 | 0.007098627 | | 0.188361337 | | 0.188361337 | |
| Phocaeicola_abscessus | | I_II | 2.096586073 | 0.704411332 | 0.003925025 | | 0.188361337 | | 0.188361337 | |
| Porphyromonas_endodontalis | | IV | 4.018084042 | 1.329824847 | 0.003865718 | | 0.188361337 | | 0.188361337 | |
| Prevotella_baroniae | | IV | 2.40569859 | 0.844498853 | 0.005877106 | | 0.188361337 | | 0.188361337 | |
| Prevotella_buccalis | | IV | 2.014983068 | 0.709031996 | 0.005808925 | | 0.188361337 | | 0.188361337 | |
| Prevotella_denticola | | IV | 3.706593313 | 1.3304186 | 0.006754805 | | 0.188361337 | | 0.188361337 | |
| Rothia_dentocariosa | | IV | 2.044106178 | 0.706632792 | 0.005002207 | | 0.188361337 | | 0.188361337 | |
| Staphylococcus_aureus | | Benign_tumor | 5.000880369 | 1.800606717 | 0.007096659 | | 0.188361337 | | 0.188361337 | |
| Treponema_amylovorum | | IV | 2.305006981 | 0.837929985 | 0.007636778 | | 0.188361337 | | 0.188361337 | |
| Rothia_dentocariosa | | I_II | 1.832701284 | 0.671632429 | 0.007956509 | | 0.189137584 | | 0.189137584 | |
| Bacteroides_caecigallinarum | | III | -2.221525448 | 0.818932601 | 0.008512172 | | 0.191494203 | | 0.191494203 | |
| Filifactor_alocis | | IV | 3.794451163 | 1.407965316 | 0.008796293 | | 0.191494203 | | 0.191494203 | |
| Haematobacter_massiliensis | | IV | -2.679590751 | 0.985616209 | 0.00880231 | | 0.191494203 | | 0.191494203 | |
| Prevotella_nanceiensis | | I_II | 2.567462093 | 0.952081604 | 0.008976291 | | 0.191494203 | | 0.191494203 | |
| Anaeroglobus_geminatus | | I_II | 2.274447494 | 0.867359294 | 0.01058392 | | 0.203547714 | | 0.203547714 | |
| Collinsella_aerofaciens | | IV | -2.920947141 | 1.114561535 | 0.010633961 | | 0.203547714 | | 0.203547714 | |
| Phocaeicola_abscessus | | IV | 1.932158624 | 0.738703906 | 0.010764543 | | 0.203547714 | | 0.203547714 | |
| Prevotella_fusca | | I_II | 3.177115636 | 1.207804694 | 0.010420195 | | 0.203547714 | | 0.203547714 | |
| Rothia_mucilaginosa | | I_II | 1.78149795 | 0.675641652 | 0.010180235 | | 0.203547714 | | 0.203547714 | |
| Anaeroglobus_geminatus | | III | 2.117238216 | 0.821266894 | 0.011921186 | | 0.206633892 | | 0.206633892 | |
| Lactiplantibacillus_plantarum | | III | -3.156137406 | 1.213839566 | 0.011238922 | | 0.206633892 | | 0.206633892 | |
| Rothia_dentocariosa | | III | 1.657569478 | 0.639759254 | 0.011497409 | | 0.206633892 | | 0.206633892 | |
| Sutterella_massiliensis | | III | 1.743190123 | 0.674035176 | 0.01167621 | | 0.206633892 | | 0.206633892 | |
| Ligilactobacillus_apodemi | | IV | -3.475022228 | 1.356598524 | 0.012489481 | | 0.207824969 | | 0.207824969 | |
| Pandoraea_pnomenusa | | I_II | -2.372563614 | 0.924999466 | 0.012383985 | | 0.207824969 | | 0.207824969 | |
| Caldicoprobacter_faecalis | | IV | -1.641144067 | 0.65100731 | 0.013856668 | | 0.215570913 | | 0.215570913 | |
| Porphyromonas_endodontalis | | III | 2.952451176 | 1.131679679 | 0.013991381 | | 0.215570913 | | 0.215570913 | |
| Propionibacterium_acidifaciens | | IV | 1.967131471 | 0.779708585 | 0.013785319 | | 0.215570913 | | 0.215570913 | |
| Sutterella_massiliensis | | I_II | 1.797892399 | 0.710308455 | 0.013498326 | | 0.215570913 | | 0.215570913 | |
| Alloscardovia_omnicolens | | III | -2.16145859 | 0.861891103 | 0.014706818 | | 0.222474041 | | 0.222474041 | |
| Bacteroides_acidifaciens | | III | -2.663255105 | 1.05093197 | 0.0153733 | | 0.225488367 | | 0.225488367 | |
| Haematobacter_massiliensis | | Benign_tumor | -2.457115773 | 0.969672206 | 0.015448121 | | 0.225488367 | | 0.225488367 | |
| Skermanella_aerolata | | I_II | 2.857490461 | 1.159030827 | 0.016011886 | | 0.229687739 | | 0.229687739 | |
| Sutterella_massiliensis | | IV | 1.828525582 | 0.748877753 | 0.017000802 | | 0.239740127 | | 0.239740127 | |
| Bifidobacterium_breve | | I_II | -2.088989776 | 0.862309328 | 0.017941927 | | 0.248794725 | | 0.248794725 | |
| Cellvibrio_mixtus | | I_II | -1.920952118 | 0.800523174 | 0.01895693 | | 0.258560092 | | 0.258560092 | |
| Brevundimonas_staleyi | | III | -2.477116256 | 1.030217122 | 0.019399855 | | 0.260333542 | | 0.260333542 | |
| Ralstonia_pickettii | | III | -3.450950338 | 1.447382595 | 0.020121975 | | 0.265737832 | | 0.265737832 | |
| Prevotella_copri | | I_II | -2.140081511 | 0.901884997 | 0.020553816 | | 0.267199607 | | 0.267199607 | |
| Herbaspirillum_huttiense | | I_II | 1.591531274 | 0.672398837 | 0.021003992 | | 0.268851101 | | 0.268851101 | |
| Pelomonas_saccharophila | | I_II | -2.731452889 | 1.165707884 | 0.02190755 | | 0.276167909 | | 0.276167909 | |
| Lactiplantibacillus_plantarum | | I_II | -2.971146779 | 1.272368555 | 0.022239928 | | 0.276173439 | | 0.276173439 | |
| Fermentimonas_caenicola | | IV | -1.691835721 | 0.728004321 | 0.022853919 | | 0.278402457 | | 0.278402457 | |
| Porphyromonas_catoniae | | I_II | 2.273366833 | 0.914091632 | 0.023088665 | | 0.278402457 | | 0.278402457 | |
| Peptoanaerobacter_stomatis | | I_II | 2.002958251 | 0.860049869 | 0.023560513 | | 0.278504564 | | 0.278504564 | |
| Slackia_exigua | | I_II | 1.956205151 | 0.847622742 | 0.023766615 | | 0.278504564 | | 0.278504564 | |
| Aggregatibacter_aphrophilus | | I_II | 3.184131926 | 1.386752982 | 0.02446458 | | 0.282701811 | | 0.282701811 | |
| Arachnia_rubra | | III | 1.657670203 | 0.726902971 | 0.025430143 | | 0.286354649 | | 0.286354649 | |
| Selenomonas_sputigena | | IV | 2.47444629 | 1.085403778 | 0.025469043 | | 0.286354649 | | 0.286354649 | |
| Acinetobacter_johnsonii | | III | -2.998070934 | 1.319775034 | 0.026166142 | | 0.286450391 | | 0.286450391 | |
| Prevotella_marshii | | III | 0.814403887 | 0.348373233 | 0.026052017 | | 0.286450391 | | 0.286450391 | |
| Acinetobacter_harbinensis | | III | -3.036835391 | 1.371258905 | 0.030077174 | | 0.287321144 | | 0.287321144 | |
| Acinetobacter_junii | | IV | -3.446148157 | 1.573651952 | 0.031657804 | | 0.287321144 | | 0.287321144 | |
| Actinomyces_dentalis | | Benign_tumor | 1.541769847 | 0.589723095 | 0.030521182 | | 0.287321144 | | 0.287321144 | |
| Actinomyces_dentalis | | III | 1.514359472 | 0.425783364 | 0.02686574 | | 0.287321144 | | 0.287321144 | |
| Aggregatibacter_aphrophilus | | Benign_tumor | 3.227017308 | 1.48064701 | 0.03243676 | | 0.287321144 | | 0.287321144 | |
| Bacteroides_caecigallinarum | | I_II | -1.898709192 | 0.870889701 | 0.032452204 | | 0.287321144 | | 0.287321144 | |
| Collinsella_aerofaciens | | I_II | -2.290807747 | 1.053181484 | 0.032807102 | | 0.287321144 | | 0.287321144 | |
| Faecalibacterium_prausnitzii | | IV | -3.003780062 | 1.368934443 | 0.031314416 | | 0.287321144 | | 0.287321144 | |
| Filifactor_alocis | | III | 2.736961329 | 1.254135674 | 0.032745496 | | 0.287321144 | | 0.287321144 | |
| Haematobacter_massiliensis | | III | -1.946179315 | 0.838718147 | 0.027653459 | | 0.287321144 | | 0.287321144 | |
| Herbaspirillum_huttiense | | III | 1.404462019 | 0.623890097 | 0.028145331 | | 0.287321144 | | 0.287321144 | |
| Lancefieldella_parvula | | III | 2.546894424 | 1.140497201 | 0.029797719 | | 0.287321144 | | 0.287321144 | |
| Olsenella_uli | | III | 2.142538143 | 0.947710337 | 0.027128467 | | 0.287321144 | | 0.287321144 | |
| Prevotella_denticola | | I_II | 2.75867115 | 1.259332549 | 0.031605343 | | 0.287321144 | | 0.287321144 | |
| Prevotella_enoeca | | I_II | 2.239056769 | 1.011430776 | 0.029910905 | | 0.287321144 | | 0.287321144 | |
| Propionibacterium_acidifaciens | | I_II | 1.643825986 | 0.741159625 | 0.029625908 | | 0.287321144 | | 0.287321144 | |
| Selenomonas_noxia | | IV | 2.203291478 | 0.984021372 | 0.028156219 | | 0.287321144 | | 0.287321144 | |
| Staphylococcus_aureus | | I_II | 3.755756812 | 1.700345613 | 0.030380297 | | 0.287321144 | | 0.287321144 | |
| Treponema_amylovorum | | III | 1.612025265 | 0.729943295 | 0.032501082 | | 0.287321144 | | 0.287321144 | |
| Phocaeicola_abscessus | | III | 1.435651946 | 0.666208605 | 0.034419183 | | 0.295727952 | | 0.295727952 | |
| Selenomonas_infelix | | IV | 1.677276387 | 0.778591877 | 0.034477898 | | 0.295727952 | | 0.295727952 | |
| Filifactor_alocis | | I_II | 2.874259801 | 1.340821233 | 0.035555093 | | 0.295818373 | | 0.295818373 | |
| Prevotella_saccharolytica | | IV | 1.553459357 | 0.723761715 | 0.035148406 | | 0.295818373 | | 0.295818373 | |
| Streptococcus_parasanguinis | | I_II | 1.181201945 | 0.534768343 | 0.035433804 | | 0.295818373 | | 0.295818373 | |
| Treponema_amylovorum | | I_II | 1.689782033 | 0.791838259 | 0.036580783 | | 0.30133873 | | 0.30133873 | |
| Streptococcus_parasanguinis | | III | 1.062415542 | 0.451458332 | 0.037453837 | | 0.305505804 | | 0.305505804 | |
| Porphyromonas_endodontalis | | I_II | 2.685802102 | 1.2672248 | 0.038831119 | | 0.313664958 | | 0.313664958 | |
| Olsenella_uli | | I_II | 2.079617537 | 1.003373839 | 0.041658009 | | 0.331882809 | | 0.331882809 | |
| Porphyromonas_gingivalis | | I_II | 2.367071343 | 1.129106708 | 0.042283146 | | 0.331882809 | | 0.331882809 | |
| Porphyromonas_gingivalis | | III | 2.147871719 | 0.999354556 | 0.042143513 | | 0.331882809 | | 0.331882809 | |
| Acinetobacter_johnsonii | | IV | -3.024045819 | 1.467962177 | 0.042891454 | | 0.333511115 | | 0.333511115 | |
| Acinetobacter_junii | | III | -2.872016129 | 1.419409763 | 0.046632356 | | 0.340248344 | | 0.340248344 | |
| Anaeroglobus_geminatus | | IV | 1.841343254 | 0.910626558 | 0.046737411 | | 0.340248344 | | 0.340248344 | |
| Bdellovibrio_exovorus | | III | -1.295449074 | 0.581215925 | 0.044878121 | | 0.340248344 | | 0.340248344 | |
| Faecalibacterium_prausnitzii | | III | -2.518956054 | 1.240418256 | 0.045827288 | | 0.340248344 | | 0.340248344 | |
| Leptotrichia_goodfellowii | | III | 1.536596233 | 0.753575943 | 0.044986821 | | 0.340248344 | | 0.340248344 | |
| Parvimonas_micra | | IV | 1.765846983 | 0.860753653 | 0.047438471 | | 0.340248344 | | 0.340248344 | |
| Prevotella_micans | | IV | 1.740848702 | 0.858815251 | 0.046213332 | | 0.340248344 | | 0.340248344 | |
| Schwartzia_succinivorans | | I_II | 1.764449118 | 0.861728653 | 0.044185414 | | 0.340248344 | | 0.340248344 | |
| Treponema_denticola | | I_II | 1.869196529 | 0.921087142 | 0.047253826 | | 0.340248344 | | 0.340248344 | |
| Bacteroides_caecigallinarum | | IV | -1.83479813 | 0.914410017 | 0.048501109 | | 0.344896775 | | 0.344896775 | |
| Phocaeicola_vulgatus | | IV | -2.482275026 | 1.240675266 | 0.049382128 | | 0.347379745 | | 0.347379745 | |
| Pseudomonas_aeruginosa | | IV | -2.015688638 | 1.006864034 | 0.049685324 | | 0.347379745 | | 0.347379745 | |

**Table S3 Differential genera and species across the five groups in duodenal fluid.**

|  | **Genus** | | | | | | |
| --- | --- | --- | --- | --- | --- | --- | --- |
| **Microbes** | | **Groups** | **coef** | **stderr** | **p-value** | **q-value** | **p-adjust** |
| Methylophilus | | III | -2.02577753 | 0.003949115 | 2.2728E-109 | 1.5E-106 | 1.5E-106 |
| Methylophilus | | I_II | -3.047473442 | 0.006839794 | 9.2501E-106 | 3.0525E-103 | 3.0525E-103 |
| Methylophilus | | IV | -2.991039307 | 0.006839843 | 2.787E-105 | 6.1314E-103 | 6.1314E-103 |
| Azospira | | III | -3.46905829 | 0.00652552 | 5.4585E-105 | 7.3402E-103 | 7.3402E-103 |
| Methylophilus | | Benign_tumor | -2.413759074 | 0.005584755 | 5.5607E-105 | 7.3402E-103 | 7.3402E-103 |
| Bacteroides | | III | -3.030854156 | 0.003644087 | 9.87783E-54 | 1.08656E-51 | 1.08656E-51 |
| Pedobacter | | III | -2.330354015 | 0.007765706 | 1.2167E-50 | 1.14718E-48 | 1.14718E-48 |
| Zoogloea | | III | -1.967589869 | 0.002101351 | 2.36687E-25 | 1.95267E-23 | 1.95267E-23 |
| Pedobacter | | IV | -3.596777182 | 0.373899724 | 1.21877E-11 | 8.93764E-10 | 8.93764E-10 |
| Bacteroides | | I_II | -3.444158891 | 0.374446243 | 1.79026E-11 | 1.18157E-09 | 1.18157E-09 |
| Pedobacter | | I_II | -3.390099587 | 0.373889112 | 5.79832E-11 | 3.47899E-09 | 3.47899E-09 |
| Bacteroides | | IV | -2.766250887 | 0.374448721 | 5.02756E-09 | 2.76516E-07 | 2.76516E-07 |
| Candidatus | | III | -2.217471116 | 0.277343693 | 1.24731E-08 | 6.33247E-07 | 6.33247E-07 |
| Candidatus | | IV | -2.204506188 | 0.317196731 | 2.38037E-08 | 1.12217E-06 | 1.12217E-06 |
| Candidatus | | I_II | -2.104028812 | 0.298003229 | 4.89084E-08 | 2.15197E-06 | 2.15197E-06 |
| Pedobacter | | Benign_tumor | -2.341428978 | 0.373839643 | 2.69101E-07 | 1.11004E-05 | 1.11004E-05 |
| Loigolactobacillus | | III | -3.214175157 | 0.632132772 | 4.1316E-06 | 0.000160403 | 0.000160403 |
| Fluviicola | | III | 0.991480716 | 0.005059813 | 2.79842E-05 | 0.001026087 | 0.001026087 |
| Loigolactobacillus | | I_II | -2.888718887 | 0.656910587 | 4.74104E-05 | 0.001646886 | 0.001646886 |
| Thermus | | III | -1.964849623 | 0.432373189 | 0.000105634 | 0.003485928 | 0.003485928 |
| Loigolactobacillus | | IV | -2.741247956 | 0.681981289 | 0.000170321 | 0.005352945 | 0.005352945 |
| Caldicoprobacter | | I_II | -2.235319324 | 0.532915136 | 0.000261573 | 0.007847195 | 0.007847195 |
| Propionibacterium | | IV | 2.970312387 | 0.775977269 | 0.000321149 | 0.00883161 | 0.00883161 |
| Pseudopedobacter | | IV | -1.892787858 | 0.491996003 | 0.000316255 | 0.00883161 | 0.00883161 |
| Alloscardovia | | I_II | -4.482932759 | 1.074547709 | 0.000357968 | 0.009353338 | 0.009353338 |
| Alloscardovia | | III | -4.18045369 | 0.990824353 | 0.000368465 | 0.009353338 | 0.009353338 |
| Azospira | | I_II | -4.698709521 | 0.263837055 | 0.000473805 | 0.011581903 | 0.011581903 |
| Pseudopedobacter | | III | -1.654042417 | 0.443848721 | 0.000523645 | 0.01234305 | 0.01234305 |
| Pandoraea | | I_II | -3.09671435 | 0.58075826 | 0.000674495 | 0.015350573 | 0.015350573 |
| Azospira | | IV | -3.905361722 | 0.263844526 | 0.000805957 | 0.01773105 | 0.01773105 |
| Caldicoprobacter | | III | -1.907520352 | 0.493714817 | 0.000847246 | 0.018038147 | 0.018038147 |
| Alloscardovia | | Benign_tumor | -4.006721938 | 1.135225619 | 0.001135097 | 0.02341137 | 0.02341137 |
| Herbaspirillum | | IV | 2.057388475 | 0.602588501 | 0.001206913 | 0.024138266 | 0.024138266 |
| Geodermatophilus | | III | -1.107562716 | 0.31456593 | 0.001492027 | 0.028962876 | 0.028962876 |
| Azospira | | Benign_tumor | -3.065037247 | 0.263778301 | 0.001618093 | 0.030512603 | 0.030512603 |
| Adlercreutzia | | I_II | -2.256095473 | 0.676117435 | 0.001665195 | 0.030528568 | 0.030528568 |
| Anaerobutyricum | | I_II | -3.303696771 | 0.692426143 | 0.002160747 | 0.038543061 | 0.038543061 |
| Faecalibacterium | | I_II | -4.340123072 | 1.036262725 | 0.00291441 | 0.049582943 | 0.049582943 |
| Thermus | | I_II | -1.514447428 | 0.468418559 | 0.002929901 | 0.049582943 | 0.049582943 |
| Olsenella | | IV | 3.463381818 | 1.106289883 | 0.003533172 | 0.057016138 | 0.057016138 |
| Propionibacterium | | I_II | 2.271187849 | 0.746468079 | 0.003541912 | 0.057016138 | 0.057016138 |
| Thauera | | I_II | -5.021797758 | 1.666451341 | 0.005834106 | 0.091678803 | 0.091678803 |
| Geodermatophilus | | I_II | -0.999193019 | 0.342532336 | 0.006526914 | 0.100180546 | 0.100180546 |
| Eggerthia | | Benign_tumor | -2.889198848 | 1.00862017 | 0.007998947 | 0.119984208 | 0.119984208 |
| Acidaminococcus | | I_II | -1.932653906 | 0.434370986 | 0.00977486 | 0.122666865 | 0.122666865 |
| Acidaminococcus | | IV | -2.02580802 | 0.451756186 | 0.009012973 | 0.122666865 | 0.122666865 |
| Atopostipes | | I_II | -1.992542854 | 0.723545663 | 0.008775888 | 0.122666865 | 0.122666865 |
| Dysgonomonas | | I_II | -1.696991407 | 0.564764474 | 0.009355532 | 0.122666865 | 0.122666865 |
| Faecalibacterium | | III | -3.870966915 | 0.887153351 | 0.009238018 | 0.122666865 | 0.122666865 |
| Geodermatophilus | | IV | -1.013845981 | 0.373236369 | 0.00999817 | 0.122666865 | 0.122666865 |
| Haemophilus | | IV | -2.364484025 | 0.879680341 | 0.01003638 | 0.122666865 | 0.122666865 |
| Mobiluncus | | I_II | -3.287767911 | 0.388347958 | 0.008934178 | 0.122666865 | 0.122666865 |
| Pandoraea | | IV | -2.031101826 | 0.60421102 | 0.009910523 | 0.122666865 | 0.122666865 |
| Thermus | | IV | -1.371307223 | 0.502556537 | 0.009690001 | 0.122666865 | 0.122666865 |
| Cellvibrio | | I_II | -1.810405587 | 0.672818936 | 0.010794909 | 0.123905001 | 0.123905001 |
| Eggerthia | | IV | -2.775794013 | 1.021893302 | 0.01117779 | 0.123905001 | 0.123905001 |
| Eubacterium | | III | -2.208495994 | 0.745621339 | 0.010666317 | 0.123905001 | 0.123905001 |
| Faucicola | | I_II | -3.851836642 | 0.802407918 | 0.011264091 | 0.123905001 | 0.123905001 |
| Peptoanaerobacter | | I_II | 3.031974395 | 1.109597971 | 0.011248603 | 0.123905001 | 0.123905001 |
| SR1_genera_incertae_sedis | | IV | -3.464209778 | 1.151382962 | 0.01082847 | 0.123905001 | 0.123905001 |
| Fusobacterium | | IV | -2.033691414 | 0.734982321 | 0.012791976 | 0.135551992 | 0.135551992 |
| Oribacterium | | III | -2.006158442 | 0.773684988 | 0.012886 | 0.135551992 | 0.135551992 |
| Rhodococcus | | I_II | 7.430406615 | 1.906754169 | 0.012939054 | 0.135551992 | 0.135551992 |
| Mobiluncus | | Benign_tumor | -2.654161749 | 0.388191719 | 0.013265269 | 0.13679809 | 0.13679809 |
| Dechloromonas | | I_II | -3.116473169 | 1.049769603 | 0.013559783 | 0.137683946 | 0.137683946 |
| Mobiluncus | | IV | -2.654108255 | 0.388366879 | 0.014565534 | 0.145655339 | 0.145655339 |
| Cellvibrio | | IV | -1.925081668 | 0.763411408 | 0.01551815 | 0.146313988 | 0.146313988 |
| Eggerthia | | III | -2.46954843 | 0.879709236 | 0.015491832 | 0.146313988 | 0.146313988 |
| Pandoraea | | III | -2.195267979 | 0.482356545 | 0.015402946 | 0.146313988 | 0.146313988 |
| Schwartzia | | I_II | 2.383936267 | 0.950021659 | 0.015107551 | 0.146313988 | 0.146313988 |
| Arachnia | | I_II | 1.5714393 | 0.569957446 | 0.015779189 | 0.146679786 | 0.146679786 |
| Sporosarcina | | I_II | -1.630584013 | 0.631396613 | 0.01623866 | 0.14885438 | 0.14885438 |
| Sutterella | | IV | 2.096065537 | 0.841477104 | 0.016863194 | 0.152461751 | 0.152461751 |
| Aliarcobacter | | I_II | -1.867821858 | 0.769575032 | 0.018376044 | 0.163894445 | 0.163894445 |
| Lacrimispora | | I_II | -2.35608136 | 0.666163388 | 0.020277194 | 0.178439307 | 0.178439307 |
| Legionella | | III | -2.665875554 | 1.001644403 | 0.02318742 | 0.200014709 | 0.200014709 |
| Solobacterium | | III | -1.665679179 | 0.706637082 | 0.023335049 | 0.200014709 | 0.200014709 |
| Anaerobutyricum | | IV | -2.122236452 | 0.803094503 | 0.02538397 | 0.214082546 | 0.214082546 |
| Escherichia.Shigella | | I_II | -4.298880776 | 1.697219338 | 0.027453883 | 0.214082546 | 0.214082546 |
| Escherichia.Shigella | | III | -4.044741168 | 1.522906674 | 0.026595686 | 0.214082546 | 0.214082546 |
| Faucicola | | Benign_tumor | -3.51480849 | 0.878521631 | 0.028045751 | 0.214082546 | 0.214082546 |
| Fudania | | Benign_tumor | -2.898888382 | 1.269531471 | 0.026086553 | 0.214082546 | 0.214082546 |
| Halomonas | | IV | 1.270147317 | 0.552131761 | 0.026943851 | 0.214082546 | 0.214082546 |
| Peptoanaerobacter | | III | 2.5655816 | 1.034163386 | 0.028219972 | 0.214082546 | 0.214082546 |
| Pseudomonas | | Benign_tumor | 4.040896921 | 1.573658777 | 0.027934741 | 0.214082546 | 0.214082546 |
| Solobacterium | | Benign_tumor | -1.762119025 | 0.774160387 | 0.027449884 | 0.214082546 | 0.214082546 |
| Sutterella | | III | 1.766068481 | 0.750650264 | 0.02711582 | 0.214082546 | 0.214082546 |
| Faucicola | | IV | -2.960265168 | 0.953877282 | 0.029005505 | 0.21754129 | 0.21754129 |
| Atopostipes | | IV | -1.688136632 | 0.751426344 | 0.03009938 | 0.221010217 | 0.221010217 |
| Howardella | | Benign_tumor | -2.760878389 | 1.242426081 | 0.030137757 | 0.221010217 | 0.221010217 |
| Actinomyces | | III | -1.438679296 | 0.65021302 | 0.030872522 | 0.221832896 | 0.221832896 |
| Halomonas | | III | 1.097812057 | 0.475252379 | 0.031207094 | 0.221832896 | 0.221832896 |
| Intestinibacter | | I_II | -2.146360764 | 0.957310946 | 0.031258272 | 0.221832896 | 0.221832896 |
| Corynebacterium | | IV | 2.211092876 | 1.00894325 | 0.032467145 | 0.225561221 | 0.225561221 |
| Pandoraea | | Benign_tumor | -1.614236138 | 0.565508634 | 0.032382289 | 0.225561221 | 0.225561221 |
| Peptostreptococcaceae_incertae_sedis | | I_II | 2.16422329 | 0.761225682 | 0.03395685 | 0.231046605 | 0.231046605 |
| Rhodococcus | | IV | 5.786344172 | 1.985558811 | 0.03389256 | 0.231046605 | 0.231046605 |
| Bacteroides | | Benign_tumor | -0.810454642 | 0.374435558 | 0.036371802 | 0.237677122 | 0.237677122 |
| Faecalibacterium | | IV | -2.888719408 | 1.212042587 | 0.036356091 | 0.237677122 | 0.237677122 |
| Massilia | | III | -2.64796537 | 1.229613527 | 0.035377452 | 0.237677122 | 0.237677122 |
| Peptostreptococcaceae_incertae_sedis | | III | 2.217074813 | 0.657334479 | 0.035784134 | 0.237677122 | 0.237677122 |
| Adlercreutzia | | III | -1.326862783 | 0.619576423 | 0.037797906 | 0.244574687 | 0.244574687 |
| Brucella | | I_II | -1.823779036 | 0.495614813 | 0.039007024 | 0.245187011 | 0.245187011 |
| Eubacterium | | Benign_tumor | -1.87377363 | 0.841678212 | 0.038589289 | 0.245187011 | 0.245187011 |
| Herbaspirillum | | III | 1.059902287 | 0.499945856 | 0.038836477 | 0.245187011 | 0.245187011 |
| Anaerobutyricum | | III | -1.837569076 | 0.602881883 | 0.040604384 | 0.25045695 | 0.25045695 |
| Sphingobium | | Benign_tumor | 2.820720215 | 1.281564187 | 0.040509616 | 0.25045695 | 0.25045695 |
| Gaiella | | I_II | -2.19526098 | 0.839714894 | 0.041997636 | 0.252589334 | 0.252589334 |
| Lactiplantibacillus | | I_II | -3.940304556 | 1.065587709 | 0.042098222 | 0.252589334 | 0.252589334 |
| Sphingobium | | III | 2.300844287 | 1.026720818 | 0.04157395 | 0.252589334 | 0.252589334 |
| Dysgonomonas | | IV | -1.311958745 | 0.590866085 | 0.042749359 | 0.254185378 | 0.254185378 |
| Lachnoanaerobaculum | | III | -1.165229059 | 0.564929141 | 0.044001758 | 0.254747019 | 0.254747019 |
| Lacrimispora | | IV | -1.95588545 | 0.69196081 | 0.04379485 | 0.254747019 | 0.254747019 |
| Peredibacter | | I_II | -3.458565482 | 0.811464739 | 0.043780363 | 0.254747019 | 0.254747019 |
| Oribacterium | | I_II | -1.69204398 | 0.824876286 | 0.045250336 | 0.259697579 | 0.259697579 |
| Butyrivibrio | | III | 2.991884225 | 1.471433032 | 0.047648199 | 0.268784714 | 0.268784714 |
| Rhodococcus | | Benign_tumor | 6.118921921 | 1.880500685 | 0.047339367 | 0.268784714 | 0.268784714 |
| Butyrivibrio | | Benign_tumor | 3.226631049 | 1.601486778 | 0.048921554 | 0.272733739 | 0.272733739 |
| Caldicoprobacter | | IV | -1.13808019 | 0.562960356 | 0.049930763 | 0.272733739 | 0.272733739 |
| Lachnospiracea_incertae_sedis | | I_II | -1.702438771 | 0.828990146 | 0.049284754 | 0.272733739 | 0.272733739 |
|  | **Species** | | | | | | |
| Prevotella_dentalis | | III | 2.230783712 | 0.003240068 | 6.5606E-117 | 5.1697E-114 | 5.1697E-114 |
| Prevotella_dentalis | | Benign_tumor | 2.230775517 | 0.004582115 | 4.9621E-108 | 1.9551E-105 | 1.9551E-105 |
| Azospira_oryzae | | III | -3.46905829 | 0.00652552 | 5.4585E-105 | 1.4338E-102 | 1.4338E-102 |
| Prevotella_dentalis | | I_II | 2.230788846 | 0.005611773 | 7.7261E-103 | 1.21961E-100 | 1.21961E-100 |
| Prevotella_dentalis | | IV | 2.230781319 | 0.005611908 | 7.7386E-103 | 1.21961E-100 | 1.21961E-100 |
| Prevotella_maculosa | | III | 1.254530927 | 0.003630667 | 3.0018E-99 | 3.94236E-97 | 3.94236E-97 |
| Prevotella_maculosa | | IV | 1.47364169 | 0.006288401 | 2.63043E-89 | 2.96111E-87 | 2.96111E-87 |
| Prevotella_maculosa | | I_II | 1.348526431 | 0.006288381 | 4.90607E-87 | 4.83248E-85 | 4.83248E-85 |
| Prevotella_maculosa | | Benign_tumor | -0.270960208 | 0.005134478 | 2.33606E-51 | 2.04535E-49 | 2.04535E-49 |
| Brevundimonas_diminuta | | III | 0.85398019 | 0.005023645 | 6.05229E-41 | 4.7692E-39 | 4.7692E-39 |
| Porphyromonas_pasteri | | III | -2.095900274 | 0.009970668 | 8.10412E-08 | 5.8055E-06 | 5.8055E-06 |
| Loigolactobacillus_coryniformis | | III | -3.214175157 | 0.632132772 | 4.1316E-06 | 0.000271309 | 0.000271309 |
| Fusobacterium_mortiferum | | III | -2.202839714 | 0.373398535 | 8.9697E-06 | 0.000543702 | 0.000543702 |
| Fusobacterium_mortiferum | | IV | -2.230190768 | 0.452846223 | 1.90291E-05 | 0.001071064 | 0.001071064 |
| Bacteroides_caecigallinarum | | III | -2.331608661 | 0.481786298 | 4.00119E-05 | 0.002101958 | 0.002101958 |
| Loigolactobacillus_coryniformis | | I_II | -2.888718887 | 0.656910587 | 4.74104E-05 | 0.00233496 | 0.00233496 |
| Fusobacterium_mortiferum | | I_II | -2.082660335 | 0.40606829 | 5.22952E-05 | 0.002424036 | 0.002424036 |
| Loigolactobacillus_coryniformis | | IV | -2.741247956 | 0.681981289 | 0.000170321 | 0.007456274 | 0.007456274 |
| Brevundimonas_diminuta | | I_II | -2.136177344 | 0.521912341 | 0.000201126 | 0.00834145 | 0.00834145 |
| Caldicoprobacter_faecalis | | I_II | -2.235319324 | 0.532915136 | 0.000261573 | 0.010305983 | 0.010305983 |
| Propionibacterium_acidifaciens | | IV | 2.970312387 | 0.775977269 | 0.000321149 | 0.012050751 | 0.012050751 |
| Alloscardovia_omnicolens | | I_II | -4.482932759 | 1.074547709 | 0.000357968 | 0.012623925 | 0.012623925 |
| Alloscardovia_omnicolens | | III | -4.18045369 | 0.990824353 | 0.000368465 | 0.012623925 | 0.012623925 |
| Azospira_oryzae | | I_II | -4.698709521 | 0.263837055 | 0.000473805 | 0.015556601 | 0.015556601 |
| Pandoraea_pnomenusa | | I_II | -3.09671435 | 0.58075826 | 0.000674495 | 0.021260079 | 0.021260079 |
| Streptococcus_mutans | | III | -3.276605821 | 0.923097534 | 0.000772739 | 0.023419922 | 0.023419922 |
| Azospira_oryzae | | IV | -3.905361722 | 0.263844526 | 0.000805957 | 0.023521999 | 0.023521999 |
| Caldicoprobacter_faecalis | | III | -1.907520352 | 0.493714817 | 0.000847246 | 0.023843932 | 0.023843932 |
| Alloscardovia_omnicolens | | Benign_tumor | -4.006721938 | 1.135225619 | 0.001135097 | 0.030843317 | 0.030843317 |
| Herbaspirillum_huttiense | | IV | 2.057388475 | 0.602588501 | 0.001206913 | 0.031701589 | 0.031701589 |
| Leptotrichia_goodfellowii | | I_II | 3.712451132 | 1.097352151 | 0.001278555 | 0.03179452 | 0.03179452 |
| Prevotella_nigrescens | | Benign_tumor | 4.517994256 | 1.336626384 | 0.001291148 | 0.03179452 | 0.03179452 |
| Prevotella_buccalis | | III | 1.510515321 | 0.39028233 | 0.00137619 | 0.032861738 | 0.032861738 |
| Bacteroides_caecigallinarum | | I_II | -1.831469255 | 0.526280247 | 0.001501166 | 0.034791728 | 0.034791728 |
| Azospira_oryzae | | Benign_tumor | -3.065037247 | 0.263778301 | 0.001618093 | 0.035418249 | 0.035418249 |
| Bifidobacterium_breve | | III | -2.731671213 | 0.767775939 | 0.001596126 | 0.035418249 | 0.035418249 |
| Adlercreutzia_muris | | I_II | -2.256095473 | 0.676117435 | 0.001665195 | 0.035464144 | 0.035464144 |
| Prevotella_buccalis | | I_II | 1.514159497 | 0.429303193 | 0.002059333 | 0.042704061 | 0.042704061 |
| Bacteroides_acidifaciens | | I_II | -4.138237595 | 1.032419938 | 0.00274942 | 0.051584359 | 0.051584359 |
| Bifidobacterium_bifidum | | IV | 2.709127545 | 0.845359882 | 0.002708363 | 0.051584359 | 0.051584359 |
| Brevundimonas_diminuta | | Benign_tumor | 1.667432742 | 0.521898079 | 0.002730837 | 0.051584359 | 0.051584359 |
| Streptococcus_mutans | | I_II | -3.004782648 | 0.959233594 | 0.002715994 | 0.051584359 | 0.051584359 |
| Faecalibacterium_prausnitzii | | I_II | -4.340123072 | 1.036262725 | 0.00291441 | 0.053408255 | 0.053408255 |
| Prevotella_buccalis | | IV | 1.547618094 | 0.483218107 | 0.003215263 | 0.05758243 | 0.05758243 |
| Olsenella_uli | | IV | 3.463381818 | 1.106289883 | 0.003533172 | 0.060674486 | 0.060674486 |
| Propionibacterium_acidifaciens | | I_II | 2.271187849 | 0.746468079 | 0.003541912 | 0.060674486 | 0.060674486 |
| Bifidobacterium_breve | | I_II | -2.509175232 | 0.827440812 | 0.004719639 | 0.079129259 | 0.079129259 |
| Capnocytophaga_granulosa | | I_II | 2.541753405 | 0.874252996 | 0.005262181 | 0.086387472 | 0.086387472 |
| Streptococcus_mutans | | Benign_tumor | -2.854719964 | 0.994628675 | 0.005716996 | 0.091938626 | 0.091938626 |
| Bdellovibrio_bacteriovorus | | I_II | -4.191329152 | 0.922806449 | 0.006051496 | 0.093501542 | 0.093501542 |
| Phocaeicola_vulgatus | | I_II | -2.964635437 | 0.976445323 | 0.006046634 | 0.093501542 | 0.093501542 |
| Prevotella_nigrescens | | III | 3.450655103 | 1.237019405 | 0.007149526 | 0.108342816 | 0.108342816 |
| Eggerthia_catenaformis | | Benign_tumor | -2.889198848 | 1.00862017 | 0.007998947 | 0.118927743 | 0.118927743 |
| Acidaminococcus_fermentans | | I_II | -1.932653906 | 0.434370986 | 0.00977486 | 0.126725443 | 0.126725443 |
| Acidaminococcus_fermentans | | IV | -2.02580802 | 0.451756186 | 0.009012973 | 0.126725443 | 0.126725443 |
| Actinomyces_graevenitzii | | IV | -3.494536681 | 1.31232242 | 0.009970784 | 0.126725443 | 0.126725443 |
| Atopostipes_suicloacalis | | I_II | -1.992542854 | 0.723545663 | 0.008775888 | 0.126725443 | 0.126725443 |
| Bdellovibrio_bacteriovorus | | IV | -3.86073916 | 1.082690811 | 0.009768925 | 0.126725443 | 0.126725443 |
| Dysgonomonas_mossii | | I_II | -1.696991407 | 0.564764474 | 0.009355532 | 0.126725443 | 0.126725443 |
| Faecalibacterium_prausnitzii | | III | -3.870966915 | 0.887153351 | 0.009238018 | 0.126725443 | 0.126725443 |
| Mobiluncus_curtisii | | I_II | -3.287767911 | 0.388347958 | 0.008934178 | 0.126725443 | 0.126725443 |
| Pandoraea_pnomenusa | | IV | -2.031101826 | 0.60421102 | 0.009910523 | 0.126725443 | 0.126725443 |
| Sphingobium_herbicidovorans | | I_II | -1.782052302 | 0.4961597 | 0.010528706 | 0.131692383 | 0.131692383 |
| Cellvibrio_mixtus | | I_II | -1.810405587 | 0.672818936 | 0.010794909 | 0.132912323 | 0.132912323 |
| Eggerthia_catenaformis | | IV | -2.775794013 | 1.021893302 | 0.01117779 | 0.134301498 | 0.134301498 |
| Peptoanaerobacter_stomatis | | I_II | 3.031974395 | 1.109597971 | 0.011248603 | 0.134301498 | 0.134301498 |
| Chryseobacterium_montanum | | I_II | -2.909734955 | 1.129642456 | 0.012818945 | 0.145915197 | 0.145915197 |
| Comamonas_sediminis | | I_II | -1.319744774 | 0.018390252 | 0.013269683 | 0.145915197 | 0.145915197 |
| Dechloromonas_agitata | | I_II | -3.116473169 | 1.049769603 | 0.013559783 | 0.145915197 | 0.145915197 |
| Haemophilus_parainfluenzae | | IV | -2.265969101 | 0.885172711 | 0.013082598 | 0.145915197 | 0.145915197 |
| Mobiluncus_curtisii | | Benign_tumor | -2.654161749 | 0.388191719 | 0.013265269 | 0.145915197 | 0.145915197 |
| Oribacterium_sinus | | III | -2.006158442 | 0.773684988 | 0.012886 | 0.145915197 | 0.145915197 |
| Prevotella_enoeca | | I_II | 3.022242367 | 1.153743783 | 0.013702696 | 0.145915197 | 0.145915197 |
| Prevotella_micans | | IV | 2.749365999 | 1.057138419 | 0.013367447 | 0.145915197 | 0.145915197 |
| Mobiluncus_curtisii | | IV | -2.654108255 | 0.388366879 | 0.014565534 | 0.153035209 | 0.153035209 |
| Cellvibrio_mixtus | | IV | -1.925081668 | 0.763411408 | 0.01551815 | 0.156773108 | 0.156773108 |
| Eggerthia_catenaformis | | III | -2.46954843 | 0.879709236 | 0.015491832 | 0.156773108 | 0.156773108 |
| Pandoraea_pnomenusa | | III | -2.195267979 | 0.482356545 | 0.015402946 | 0.156773108 | 0.156773108 |
| Arachnia_rubra | | I_II | 1.5714393 | 0.569957446 | 0.015779189 | 0.157392418 | 0.157392418 |
| Sporosarcina_globispora | | I_II | -1.630584013 | 0.631396613 | 0.01623866 | 0.159950797 | 0.159950797 |
| Bifidobacterium_bifidum | | I_II | 2.036381644 | 0.799428601 | 0.016840193 | 0.162051178 | 0.162051178 |
| Sutterella_massiliensis | | IV | 2.096065537 | 0.841477104 | 0.016863194 | 0.162051178 | 0.162051178 |
| Bifidobacterium_bifidum | | III | 1.930235077 | 0.742553979 | 0.017151818 | 0.162838943 | 0.162838943 |
| Aliarcobacter_cryaerophilus | | I_II | -1.867821858 | 0.769575032 | 0.018376044 | 0.172384792 | 0.172384792 |
| Prevotella_micans | | I_II | 2.501430582 | 1.017860658 | 0.01896687 | 0.175834038 | 0.175834038 |
| Neisseria_oralis | | I_II | 3.059048328 | 0.94457092 | 0.019302795 | 0.176867467 | 0.176867467 |
| Lacrimispora_sphenoides | | I_II | -2.35608136 | 0.666163388 | 0.020277194 | 0.181573055 | 0.181573055 |
| Treponema_amylovorum | | III | 2.555898504 | 1.025215539 | 0.020090647 | 0.181573055 | 0.181573055 |
| Capnocytophaga_sputigena | | I_II | 3.136654227 | 1.334212263 | 0.022290684 | 0.197360211 | 0.197360211 |
| Solobacterium_moorei | | III | -1.665679179 | 0.706637082 | 0.023335049 | 0.204311322 | 0.204311322 |
| Bdellovibrio_bacteriovorus | | Benign_tumor | -3.334554724 | 1.021253076 | 0.025727742 | 0.222785279 | 0.222785279 |
| Escherichia.Shigella_coli | | I_II | -4.298880776 | 1.697219338 | 0.027453883 | 0.225453934 | 0.225453934 |
| Escherichia.Shigella_coli | | III | -4.044741168 | 1.522906674 | 0.026595686 | 0.225453934 | 0.225453934 |
| Solobacterium_moorei | | Benign_tumor | -1.762119025 | 0.774160387 | 0.027449884 | 0.225453934 | 0.225453934 |
| Sutterella_massiliensis | | III | 1.766068481 | 0.750650264 | 0.02711582 | 0.225453934 | 0.225453934 |
| Treponema_amylovorum | | I_II | 2.528485057 | 1.102583517 | 0.027466469 | 0.225453934 | 0.225453934 |
| Peptoanaerobacter_stomatis | | III | 2.5655816 | 1.034163386 | 0.028219972 | 0.229250906 | 0.229250906 |
| Atopostipes_suicloacalis | | IV | -1.688136632 | 0.751426344 | 0.03009938 | 0.235134182 | 0.235134182 |
| Bacteroides_caecigallinarum | | IV | -1.308019195 | 0.578417364 | 0.029778806 | 0.235134182 | 0.235134182 |
| Campylobacter_gracilis | | III | 2.285823815 | 1.025273084 | 0.029666898 | 0.235134182 | 0.235134182 |
| Howardella_ureilytica | | Benign_tumor | -2.760878389 | 1.242426081 | 0.030137757 | 0.235134182 | 0.235134182 |
| Schwartzia_succinivorans | | I_II | 2.062962299 | 0.903330051 | 0.031341012 | 0.242124679 | 0.242124679 |
| Prevotella_micans | | III | 2.162433956 | 0.943533393 | 0.032054192 | 0.24523013 | 0.24523013 |
| Pandoraea_pnomenusa | | Benign_tumor | -1.614236138 | 0.565508634 | 0.032382289 | 0.245358112 | 0.245358112 |
| Eubacterium_brachy | | Benign_tumor | -2.696024467 | 1.233630594 | 0.034003591 | 0.250802686 | 0.250802686 |
| Phocaeicola_vulgatus | | III | -2.172054703 | 0.899790672 | 0.034055695 | 0.250802686 | 0.250802686 |
| Treponema_lecithinolyticum | | I_II | 1.687346536 | 0.746389014 | 0.033666135 | 0.250802686 | 0.250802686 |
| Actinomyces_massiliensis | | Benign_tumor | -2.676073995 | 1.194458552 | 0.034468064 | 0.251489205 | 0.251489205 |
| Bacteroides_acidifaciens | | III | -2.719305774 | 0.882938638 | 0.036071365 | 0.260441813 | 0.260441813 |
| Faecalibacterium_prausnitzii | | IV | -2.888719408 | 1.212042587 | 0.036356091 | 0.260441813 | 0.260441813 |
| Sphingobium_herbicidovorans | | Benign_tumor | -1.48928607 | 0.550414672 | 0.036803375 | 0.261270805 | 0.261270805 |
| Adlercreutzia_muris | | III | -1.326862783 | 0.619576423 | 0.037797906 | 0.265935269 | 0.265935269 |
| Bdellovibrio_bacteriovorus | | III | -2.980315932 | 0.787317091 | 0.039085392 | 0.2701692 | 0.2701692 |
| Herbaspirillum_huttiense | | III | 1.059902287 | 0.499945856 | 0.038836477 | 0.2701692 | 0.2701692 |
| Prevotella_nigrescens | | I_II | 2.702584181 | 1.288377494 | 0.040233126 | 0.275684379 | 0.275684379 |
| Dysgonomonas_mossii | | IV | -1.311958745 | 0.590866085 | 0.042749359 | 0.277387081 | 0.277387081 |
| Gaiella_occulta | | I_II | -2.19526098 | 0.839714894 | 0.041997636 | 0.277387081 | 0.277387081 |
| Lachnoanaerobaculum_umeaense | | III | -1.165229059 | 0.564929141 | 0.044001758 | 0.277387081 | 0.277387081 |
| Lacrimispora_sphenoides | | IV | -1.95588545 | 0.69196081 | 0.04379485 | 0.277387081 | 0.277387081 |
| Lactiplantibacillus_plantarum | | I_II | -3.940304556 | 1.065587709 | 0.042098222 | 0.277387081 | 0.277387081 |
| Leptotrichia_goodfellowii | | III | 2.189199836 | 1.056120872 | 0.042569208 | 0.277387081 | 0.277387081 |
| Peredibacter_starrii | | I_II | -3.458565482 | 0.811464739 | 0.043780363 | 0.277387081 | 0.277387081 |
| Prevotella_denticola | | III | 3.536938321 | 1.697619679 | 0.041549847 | 0.277387081 | 0.277387081 |
| Prevotella_nanceiensis | | IV | -2.060536533 | 0.932164244 | 0.043534658 | 0.277387081 | 0.277387081 |
| Schaalia_georgiae | | III | -2.39816907 | 1.147019072 | 0.041478275 | 0.277387081 | 0.277387081 |
| Bifidobacterium_breve | | IV | -1.793120402 | 0.870083902 | 0.045493816 | 0.282276591 | 0.282276591 |
| Oribacterium_sinus | | I_II | -1.69204398 | 0.824876286 | 0.045250336 | 0.282276591 | 0.282276591 |
| Streptococcus_salivarius | | I_II | -4.787194646 | 2.358386054 | 0.046965405 | 0.289130774 | 0.289130774 |
| Caldicoprobacter_faecalis | | IV | -1.13808019 | 0.562960356 | 0.049930763 | 0.305003418 | 0.305003418 |

**Table S4 Differential genera and species across the five groups in saliva.**

|  | **Genus** | | | | | | | | | |
| --- | --- | --- | --- | --- | --- | --- | --- | --- | --- | --- |
| **Microbes** | | **Groups** | **coef** | **stderr** | | **p-value** | | **q-value** | | **p-adjust** |
| Olsenella | | I_II | 3.965623938 | | 0.845313234 | | 1.19408E-05 | | 0.007403304 | 0.007403304 |
| Lactiplantibacillus | | I_II | -4.512613758 | | 1.038299176 | | 4.54016E-05 | | 0.008103877 | 0.008103877 |
| Lactiplantibacillus | | III | -4.235210357 | | 0.959922212 | | 4.58765E-05 | | 0.008103877 | 0.008103877 |
| Loigolactobacillus | | III | -1.932552523 | | 0.44992965 | | 5.22831E-05 | | 0.008103877 | 0.008103877 |
| Arachnia | | I_II | 3.553984744 | | 0.846602037 | | 7.39896E-05 | | 0.008342057 | 0.008342057 |
| Loigolactobacillus | | IV | -2.084761064 | | 0.499497582 | | 8.07296E-05 | | 0.008342057 | 0.008342057 |
| Olsenella | | III | 3.319146245 | | 0.807263074 | | 9.95241E-05 | | 0.008814993 | 0.008814993 |
| Peptoanaerobacter | | I_II | 3.645624735 | | 0.91379495 | | 0.000154548 | | 0.011977446 | 0.011977446 |
| Olsenella | | IV | 3.360847364 | | 0.890901292 | | 0.000320727 | | 0.022094555 | 0.022094555 |
| Lactiplantibacillus | | IV | -4.084259691 | | 1.098513257 | | 0.000395555 | | 0.02452443 | 0.02452443 |
| Abiotrophia | | I_II | 3.996004041 | | 1.119530062 | | 0.000648902 | | 0.036574455 | 0.036574455 |
| Akkermansia | | I_II | -1.302040071 | | 0.373330204 | | 0.000837058 | | 0.043247976 | 0.043247976 |
| Akkermansia | | III | -1.158203728 | | 0.346234972 | | 0.001414844 | | 0.062657359 | 0.062657359 |
| Atopostipes | | I_II | -1.202177098 | | 0.360941787 | | 0.001361476 | | 0.062657359 | 0.062657359 |
| Atopostipes | | III | -1.106605014 | | 0.3373187 | | 0.001666604 | | 0.068886295 | 0.068886295 |
| Peptostreptococcaceae_incertae_sedis | | III | 2.717552647 | | 0.74515041 | | 0.002310484 | | 0.089531257 | 0.089531257 |
| Peptoanaerobacter | | III | 2.70978925 | | 0.863324601 | | 0.002546071 | | 0.089828516 | 0.089828516 |
| Propionibacterium | | I_II | 2.770458207 | | 0.889149411 | | 0.002607925 | | 0.089828516 | 0.089828516 |
| Streptobacillus | | I_II | 1.980459073 | | 0.640684965 | | 0.002819336 | | 0.09199938 | 0.09199938 |
| Peredibacter | | III | -1.058055804 | | 0.340680981 | | 0.003260636 | | 0.096266411 | 0.096266411 |
| Sutterella | | I_II | 2.147930871 | | 0.702763472 | | 0.003107231 | | 0.096266411 | 0.096266411 |
| Brevundimonas | | I_II | -2.453810697 | | 0.826124194 | | 0.004026665 | | 0.108544893 | 0.108544893 |
| Loigolactobacillus | | I_II | -1.404244472 | | 0.471738556 | | 0.003934674 | | 0.108544893 | 0.108544893 |
| Anaerobutyricum | | I_II | -1.199318708 | | 0.405986969 | | 0.004230289 | | 0.109282468 | 0.109282468 |
| Dolosigranulum | | I_II | 2.598715973 | | 0.885752892 | | 0.004444444 | | 0.110222214 | 0.110222214 |
| Blautia | | I_II | -1.815418773 | | 0.628997678 | | 0.005262764 | | 0.125496678 | 0.125496678 |
| Abiotrophia | | Benign_tumor | 3.275867627 | | 1.183611933 | | 0.007379865 | | 0.131456875 | 0.131456875 |
| Anaerobutyricum | | III | -1.077149825 | | 0.376949556 | | 0.005781444 | | 0.131456875 | 0.131456875 |
| Anaeroglobus | | III | 2.304772888 | | 0.811347128 | | 0.006566131 | | 0.131456875 | 0.131456875 |
| Azonexus | | I_II | -1.404216945 | | 0.502499924 | | 0.006793755 | | 0.131456875 | 0.131456875 |
| Azospira | | III | -1.334041326 | | 0.477416653 | | 0.007416051 | | 0.131456875 | 0.131456875 |
| Bacteroides | | III | -1.427538519 | | 0.511167761 | | 0.007420953 | | 0.131456875 | 0.131456875 |
| Faecalibacterium | | I_II | -2.328346199 | | 0.833432789 | | 0.0068042 | | 0.131456875 | 0.131456875 |
| Peptostreptococcaceae_incertae_sedis | | I_II | 2.53762185 | | 0.873238852 | | 0.006331038 | | 0.131456875 | 0.131456875 |
| Schaalia | | IV | -1.175370579 | | 0.413283624 | | 0.006666003 | | 0.131456875 | 0.131456875 |
| Peredibacter | | I_II | -1.042085399 | | 0.380555931 | | 0.008146966 | | 0.140308856 | 0.140308856 |
| Arachnia | | III | 2.16228423 | | 0.799891943 | | 0.008561342 | | 0.143460324 | 0.143460324 |
| Faecalibacterium | | III | -2.054151767 | | 0.754436348 | | 0.008954712 | | 0.146103191 | 0.146103191 |
| Caldicoprobacter | | I_II | -1.556123348 | | 0.586450613 | | 0.009869951 | | 0.15242207 | 0.15242207 |
| Caldicoprobacter | | III | -1.448398362 | | 0.545775939 | | 0.010079524 | | 0.15242207 | 0.15242207 |
| Halomonas | | I_II | 1.481214931 | | 0.557027017 | | 0.009596791 | | 0.15242207 | 0.15242207 |
| Adlercreutzia | | I_II | -0.868977329 | | 0.3364997 | | 0.011853949 | | 0.152971831 | 0.152971831 |
| Akkermansia | | IV | -1.024041976 | | 0.396412915 | | 0.011798525 | | 0.152971831 | 0.152971831 |
| Anaeroglobus | | I_II | 2.232558499 | | 0.87857645 | | 0.013327747 | | 0.152971831 | 0.152971831 |
| Atopostipes | | IV | -0.975195269 | | 0.383185351 | | 0.013023459 | | 0.152971831 | 0.152971831 |
| Blautia | | III | -1.474444851 | | 0.571032157 | | 0.012899823 | | 0.152971831 | 0.152971831 |
| Brevundimonas | | III | -1.985148718 | | 0.765479636 | | 0.011931127 | | 0.152971831 | 0.152971831 |
| Bulleidia | | I_II | 2.009181642 | | 0.792933835 | | 0.013471264 | | 0.152971831 | 0.152971831 |
| Bulleidia | | III | 1.930308016 | | 0.741758919 | | 0.011928353 | | 0.152971831 | 0.152971831 |
| Cellvibrio | | I_II | -0.853062597 | | 0.325170872 | | 0.010590433 | | 0.152971831 | 0.152971831 |
| Delftia | | I_II | -2.411116258 | | 0.932231285 | | 0.011696008 | | 0.152971831 | 0.152971831 |
| Fluviicola | | I_II | -0.876562579 | | 0.343309659 | | 0.012828247 | | 0.152971831 | 0.152971831 |
| Ligilactobacillus | | IV | -1.61905049 | | 0.612516476 | | 0.012591761 | | 0.152971831 | 0.152971831 |
| Peptoanaerobacter | | IV | 2.437267157 | | 0.96371608 | | 0.013570082 | | 0.152971831 | 0.152971831 |
| Solobacterium | | IV | -1.680313427 | | 0.662883006 | | 0.013533489 | | 0.152971831 | 0.152971831 |
| Azonexus | | III | -1.160852088 | | 0.457164695 | | 0.014139332 | | 0.156542606 | 0.156542606 |
| Azospira | | I_II | -1.307408621 | | 0.523439591 | | 0.014977671 | | 0.16010614 | 0.16010614 |
| Geodermatophilus | | III | -1.455211867 | | 0.583584325 | | 0.014850144 | | 0.16010614 | 0.16010614 |
| Limnobacter | | I_II | 1.67337943 | | 0.676421855 | | 0.01567164 | | 0.162137559 | 0.162137559 |
| Stenotrophomonas | | I_II | -1.357867862 | | 0.547081882 | | 0.015690731 | | 0.162137559 | 0.162137559 |
| Slackia | | I_II | 2.157718444 | | 0.884098005 | | 0.018379234 | | 0.186805327 | 0.186805327 |
| Halomonas | | III | 1.251973776 | | 0.526870942 | | 0.020158635 | | 0.201586351 | 0.201586351 |
| Herbaspirillum | | IV | 1.756541221 | | 0.745887256 | | 0.021186429 | | 0.20850136 | 0.20850136 |
| Dysgonomonas | | I_II | -0.778280351 | | 0.33156966 | | 0.021663032 | | 0.209860627 | 0.209860627 |
| Herbaspirillum | | I_II | 1.6368027 | | 0.704955334 | | 0.023145696 | | 0.220774333 | 0.220774333 |
| Aminivibrio | | I_II | 1.420030062 | | 0.614938644 | | 0.023855948 | | 0.224101331 | 0.224101331 |
| Anaeroglobus | | IV | 2.120245749 | | 0.92709933 | | 0.025236218 | | 0.230009553 | 0.230009553 |
| Herbaspirillum | | III | 1.535749408 | | 0.671923407 | | 0.025146201 | | 0.230009553 | 0.230009553 |
| Peredibacter | | IV | -0.908591719 | | 0.396610461 | | 0.025933527 | | 0.230009553 | 0.230009553 |
| Schwartzia | | I_II | 2.511850183 | | 1.103844215 | | 0.025968821 | | 0.230009553 | 0.230009553 |
| Halomonas | | IV | 1.325926052 | | 0.586482826 | | 0.026729493 | | 0.232301162 | 0.232301162 |
| Sutterella | | IV | 1.66455509 | | 0.737791873 | | 0.026976909 | | 0.232301162 | 0.232301162 |
| Geodermatophilus | | I_II | -1.372005098 | | 0.611091438 | | 0.027704119 | | 0.235295256 | 0.235295256 |
| Fluviicola | | III | -0.710701708 | | 0.317525299 | | 0.028778079 | | 0.241113635 | 0.241113635 |
| Abiotrophia | | III | 2.300791793 | | 1.038702172 | | 0.030972572 | | 0.256039926 | 0.256039926 |
| Capnocytophaga | | I_II | 1.723187615 | | 0.788475018 | | 0.031998921 | | 0.261043829 | 0.261043829 |
| Adlercreutzia | | III | -0.676125809 | | 0.3093278 | | 0.032669789 | | 0.263055443 | 0.263055443 |
| Streptobacillus | | III | 1.302904696 | | 0.603427958 | | 0.034266598 | | 0.268954121 | 0.268954121 |
| Sutterella | | III | 1.436159763 | | 0.665740507 | | 0.034269961 | | 0.268954121 | 0.268954121 |
| Azospira | | IV | -1.174466325 | | 0.554246861 | | 0.037779561 | | 0.279384943 | 0.279384943 |
| Delftia | | III | -1.844871639 | | 0.863874314 | | 0.036650643 | | 0.279384943 | 0.279384943 |
| Geodermatophilus | | IV | -1.362768338 | | 0.644047827 | | 0.037668808 | | 0.279384943 | 0.279384943 |
| Solobacterium | | III | -1.229055625 | | 0.578661179 | | 0.037852154 | | 0.279384943 | 0.279384943 |
| Thermus | | III | -0.999713049 | | 0.460413031 | | 0.037359047 | | 0.279384943 | 0.279384943 |
| Lancefieldella | | I_II | -1.93157569 | | 0.920745854 | | 0.039288195 | | 0.286572716 | 0.286572716 |
| Aliarcobacter | | III | -1.096462204 | | 0.511800667 | | 0.041113548 | | 0.296399999 | 0.296399999 |
| Bradyrhizobium | | I_II | 2.73424566 | | 1.319297954 | | 0.041736163 | | 0.297430127 | 0.297430127 |
| Faecalibacterium | | IV | -1.80943534 | | 0.87382264 | | 0.042398091 | | 0.298713826 | 0.298713826 |
| Aminivibrio | | III | 1.183665543 | | 0.572704898 | | 0.042947998 | | 0.299188304 | 0.299188304 |
| Brevundimonas | | IV | -1.759784244 | | 0.868367966 | | 0.046391317 | | 0.319584631 | 0.319584631 |
| Caldicoprobacter | | IV | -1.244405891 | | 0.616396703 | | 0.047356227 | | 0.322646821 | 0.322646821 |
| Johnsonella | | I_II | 1.232077382 | | 0.613727671 | | 0.049646219 | | 0.334572345 | 0.334572345 |
|  | **Species** | | | | | | | | | |
| Fusobacterium_mortiferum | | III | -2.047659415 | 0.395199523 | | 3.52151E-06 | | 0.002605918 | | 0.002605918 |
| Fusobacterium_mortiferum | | IV | -2.137465808 | 0.451385187 | | 1.26786E-05 | | 0.003127394 | | 0.003127394 |
| Olsenella_uli | | I_II | 3.965623938 | 0.845313234 | | 1.19408E-05 | | 0.003127394 | | 0.003127394 |
| Lactiplantibacillus_plantarum | | I_II | -4.512613758 | 1.038299176 | | 4.54016E-05 | | 0.006448246 | | 0.006448246 |
| Lactiplantibacillus_plantarum | | III | -4.235210357 | 0.959922212 | | 4.58765E-05 | | 0.006448246 | | 0.006448246 |
| Loigolactobacillus_coryniformis | | III | -1.932552523 | 0.44992965 | | 5.22831E-05 | | 0.006448246 | | 0.006448246 |
| Prevotella_buccalis | | I_II | 2.40576505 | 0.565782433 | | 6.10468E-05 | | 0.006453515 | | 0.006453515 |
| Arachnia_rubra | | I_II | 3.553984744 | 0.846602037 | | 7.39896E-05 | | 0.006637766 | | 0.006637766 |
| Loigolactobacillus_coryniformis | | IV | -2.084761064 | 0.499497582 | | 8.07296E-05 | | 0.006637766 | | 0.006637766 |
| Olsenella_uli | | III | 3.319146245 | 0.807263074 | | 9.95241E-05 | | 0.007364785 | | 0.007364785 |
| Peptoanaerobacter_stomatis | | I_II | 3.645624735 | 0.91379495 | | 0.000154548 | | 0.010396845 | | 0.010396845 |
| Phocaeicola_abscessus | | I_II | 3.30855973 | 0.856723796 | | 0.000236857 | | 0.014606202 | | 0.014606202 |
| Olsenella_uli | | IV | 3.360847364 | 0.890901292 | | 0.000320727 | | 0.018256791 | | 0.018256791 |
| Brevundimonas_diminuta | | I_II | -2.862029719 | 0.770500909 | | 0.000405635 | | 0.018760612 | | 0.018760612 |
| Fusobacterium_mortiferum | | I_II | -1.609983716 | 0.429951756 | | 0.000392952 | | 0.018760612 | | 0.018760612 |
| Lactiplantibacillus_plantarum | | IV | -4.084259691 | 1.098513257 | | 0.000395555 | | 0.018760612 | | 0.018760612 |
| Corynebacterium_pseudodiphtheriticum | | I_II | 3.382425974 | 0.732085839 | | 0.000534219 | | 0.023254244 | | 0.023254244 |
| Abiotrophia_defectiva | | I_II | 3.996004041 | 1.119530062 | | 0.000648902 | | 0.026677066 | | 0.026677066 |
| Bifidobacterium_bifidum | | I_II | 2.41275947 | 0.689154424 | | 0.00079201 | | 0.030846686 | | 0.030846686 |
| Akkermansia_muciniphila | | I_II | -1.302040071 | 0.373330204 | | 0.000837058 | | 0.030971131 | | 0.030971131 |
| Brevundimonas_diminuta | | III | -2.466124231 | 0.70404988 | | 0.000899728 | | 0.031704714 | | 0.031704714 |
| Phocaeicola_vulgatus | | I_II | -1.777262634 | 0.519119753 | | 0.001019776 | | 0.03281018 | | 0.03281018 |
| Prevotella_buccalis | | III | 1.848318274 | 0.539626371 | | 0.001005387 | | 0.03281018 | | 0.03281018 |
| Phocaeicola_vulgatus | | III | -1.63653716 | 0.482955616 | | 0.001212163 | | 0.037375018 | | 0.037375018 |
| Akkermansia_muciniphila | | III | -1.158203728 | 0.346234972 | | 0.001414844 | | 0.038943657 | | 0.038943657 |
| Atopostipes_suicloacalis | | I_II | -1.202177098 | 0.360941787 | | 0.001361476 | | 0.038943657 | | 0.038943657 |
| Bacteroides_acidifaciens | | III | -1.367940167 | 0.386607657 | | 0.00152617 | | 0.038943657 | | 0.038943657 |
| Leptotrichia_goodfellowii | | I_II | 3.519770709 | 1.066272056 | | 0.001476741 | | 0.038943657 | | 0.038943657 |
| Prevotella_pallens | | I_II | -5.890959516 | 1.783310706 | | 0.001468368 | | 0.038943657 | | 0.038943657 |
| Atopostipes_suicloacalis | | III | -1.106605014 | 0.3373187 | | 0.001666604 | | 0.041109563 | | 0.041109563 |
| Haemophilus_aegyptius | | I_II | 3.786039886 | 1.146718844 | | 0.001752511 | | 0.041834128 | | 0.041834128 |
| Neisseria_oralis | | I_II | 3.590510026 | 1.112029804 | | 0.001882664 | | 0.042217318 | | 0.042217318 |
| Prevotella_copri | | I_II | -1.216730653 | 0.375222532 | | 0.001850231 | | 0.042217318 | | 0.042217318 |
| Capnocytophaga_sputigena | | I_II | 3.036395564 | 0.947779115 | | 0.001992654 | | 0.043369519 | | 0.043369519 |
| Prevotella_buccalis | | IV | 1.906115354 | 0.599073057 | | 0.002139571 | | 0.045236645 | | 0.045236645 |
| Prevotella_denticola | | IV | 3.223048859 | 1.019876745 | | 0.002275645 | | 0.046777145 | | 0.046777145 |
| Peptoanaerobacter_stomatis | | III | 2.70978925 | 0.863324601 | | 0.002546071 | | 0.050785901 | | 0.050785901 |
| Propionibacterium_acidifaciens | | I_II | 2.770458207 | 0.889149411 | | 0.002607925 | | 0.050785901 | | 0.050785901 |
| Prevotella_denticola | | I_II | 3.010366695 | 0.970095439 | | 0.002710952 | | 0.051438579 | | 0.051438579 |
| Sutterella_massiliensis | | I_II | 2.147930871 | 0.702763472 | | 0.003107231 | | 0.057483764 | | 0.057483764 |
| Peredibacter_starrii | | III | -1.058055804 | 0.340680981 | | 0.003260636 | | 0.058850512 | | 0.058850512 |
| Loigolactobacillus_coryniformis | | I_II | -1.404244472 | 0.471738556 | | 0.003934674 | | 0.069325208 | | 0.069325208 |
| Prevotella_copri | | III | -1.009825259 | 0.339049616 | | 0.00431818 | | 0.074312862 | | 0.074312862 |
| Dolosigranulum_pigrum | | I_II | 2.598715973 | 0.885752892 | | 0.004444444 | | 0.074747469 | | 0.074747469 |
| Anaeroglobus_geminatus | | III | 2.304772888 | 0.811347128 | | 0.006566131 | | 0.102909311 | | 0.102909311 |
| Corynebacterium_pseudodiphtheriticum | | III | 2.613169106 | 0.628314796 | | 0.006814265 | | 0.102909311 | | 0.102909311 |
| Faecalibacterium_prausnitzii | | I_II | -2.328346199 | 0.833432789 | | 0.0068042 | | 0.102909311 | | 0.102909311 |
| Prevotella_denticola | | III | 2.568359188 | 0.922870954 | | 0.006810747 | | 0.102909311 | | 0.102909311 |
| Prevotella_enoeca | | I_II | 3.046441913 | 1.08970872 | | 0.006626523 | | 0.102909311 | | 0.102909311 |
| Abiotrophia_defectiva | | Benign_tumor | 3.275867627 | 1.183611933 | | 0.007379865 | | 0.105536112 | | 0.105536112 |
| Azospira_oryzae | | III | -1.334041326 | 0.477416653 | | 0.007416051 | | 0.105536112 | | 0.105536112 |
| Leptotrichia_goodfellowii | | III | 2.807455865 | 1.018275857 | | 0.007320109 | | 0.105536112 | | 0.105536112 |
| Bacteroides_acidifaciens | | IV | -1.273087444 | 0.46048278 | | 0.007862014 | | 0.10977152 | | 0.10977152 |
| Peredibacter_starrii | | I_II | -1.042085399 | 0.380555931 | | 0.008146966 | | 0.111643606 | | 0.111643606 |
| Arachnia_rubra | | III | 2.16228423 | 0.799891943 | | 0.008561342 | | 0.114729984 | | 0.114729984 |
| Schaalia_odontolytica | | IV | -1.28201402 | 0.466955109 | | 0.008682269 | | 0.114729984 | | 0.114729984 |
| Bacteroides_acidifaciens | | I_II | -1.180973999 | 0.432979685 | | 0.009007483 | | 0.114923058 | | 0.114923058 |
| Faecalibacterium_prausnitzii | | III | -2.054151767 | 0.754436348 | | 0.008954712 | | 0.114923058 | | 0.114923058 |
| Veillonella_dispar | | I_II | 1.016521038 | 0.380045496 | | 0.009176289 | | 0.115092442 | | 0.115092442 |
| Blautia_luti | | III | -0.965791592 | 0.363788167 | | 0.010184087 | | 0.121552006 | | 0.121552006 |
| Caldicoprobacter_faecalis | | I_II | -1.556123348 | 0.586450613 | | 0.009869951 | | 0.121552006 | | 0.121552006 |
| Caldicoprobacter_faecalis | | III | -1.448398362 | 0.545775939 | | 0.010079524 | | 0.121552006 | | 0.121552006 |
| Alloprevotella_rava | | I_II | 2.708065536 | 1.032822502 | | 0.010664011 | | 0.123302632 | | 0.123302632 |
| Cellvibrio_mixtus | | I_II | -0.853062597 | 0.325170872 | | 0.010590433 | | 0.123302632 | | 0.123302632 |
| Schwartzia_succinivorans | | I_II | 2.605060255 | 0.993993998 | | 0.010886799 | | 0.12394202 | | 0.12394202 |
| Adlercreutzia_muris | | I_II | -0.868977329 | 0.3364997 | | 0.011853949 | | 0.125523257 | | 0.125523257 |
| Akkermansia_muciniphila | | IV | -1.024041976 | 0.396412915 | | 0.011798525 | | 0.125523257 | | 0.125523257 |
| Alloprevotella_rava | | III | 2.45101369 | 0.967031078 | | 0.013533812 | | 0.125523257 | | 0.125523257 |
| Anaeroglobus_geminatus | | I_II | 2.232558499 | 0.87857645 | | 0.013327747 | | 0.125523257 | | 0.125523257 |
| Atopostipes_suicloacalis | | IV | -0.975195269 | 0.383185351 | | 0.013023459 | | 0.125523257 | | 0.125523257 |
| Brevundimonas_diminuta | | IV | -2.07849972 | 0.807594387 | | 0.012242977 | | 0.125523257 | | 0.125523257 |
| Bulleidia_extructa | | I_II | 2.009181642 | 0.792933835 | | 0.013471264 | | 0.125523257 | | 0.125523257 |
| Bulleidia_extructa | | III | 1.930308016 | 0.741758919 | | 0.011928353 | | 0.125523257 | | 0.125523257 |
| Delftia_tsuruhatensis | | I_II | -2.411116258 | 0.932231285 | | 0.011696008 | | 0.125523257 | | 0.125523257 |
| Peptoanaerobacter_stomatis | | IV | 2.437267157 | 0.96371608 | | 0.013570082 | | 0.125523257 | | 0.125523257 |
| Phocaeicola_vulgatus | | IV | -1.405923645 | 0.550843252 | | 0.012779787 | | 0.125523257 | | 0.125523257 |
| Prevotella_dentalis | | I_II | 2.357859551 | 0.930431304 | | 0.013369455 | | 0.125523257 | | 0.125523257 |
| Prevotella_micans | | I_II | 2.059766482 | 0.810844656 | | 0.013149101 | | 0.125523257 | | 0.125523257 |
| Pseudomonas_aeruginosa | | I_II | -1.374480241 | 0.528214839 | | 0.011290271 | | 0.125523257 | | 0.125523257 |
| Solobacterium_moorei | | IV | -1.680313427 | 0.662883006 | | 0.013533489 | | 0.125523257 | | 0.125523257 |
| Ligilactobacillus_apodemi | | IV | -1.635474011 | 0.630601864 | | 0.014059611 | | 0.12844583 | | 0.12844583 |
| Phocaeicola_abscessus | | III | 2.042635219 | 0.818160011 | | 0.014733181 | | 0.132957973 | | 0.132957973 |
| Azospira_oryzae | | I_II | -1.307408621 | 0.523439591 | | 0.014977671 | | 0.133535863 | | 0.133535863 |
| Limnobacter_thiooxidans | | I_II | 1.67337943 | 0.676421855 | | 0.01567164 | | 0.136601662 | | 0.136601662 |
| Stenotrophomonas_maltophilia | | I_II | -1.357867862 | 0.547081882 | | 0.015690731 | | 0.136601662 | | 0.136601662 |
| Corynebacterium_pseudodiphtheriticum | | Benign_tumor | 2.152146827 | 0.775697156 | | 0.016256795 | | 0.13988405 | | 0.13988405 |
| Neisseria_oralis | | III | 2.526433584 | 1.044439658 | | 0.018497457 | | 0.155546794 | | 0.155546794 |
| Slackia_exigua | | I_II | 2.157718444 | 0.884098005 | | 0.018379234 | | 0.155546794 | | 0.155546794 |
| Metamycoplasma_salivarium | | I_II | 1.646309604 | 0.689738502 | | 0.019614339 | | 0.163085518 | | 0.163085518 |
| Actinomyces_graevenitzii | | I_II | -2.455436652 | 1.042159432 | | 0.021213416 | | 0.171533147 | | 0.171533147 |
| Dysgonomonas_mossii | | I_II | -0.778280351 | 0.33156966 | | 0.021663032 | | 0.171533147 | | 0.171533147 |
| Herbaspirillum_huttiense | | IV | 1.756541221 | 0.745887256 | | 0.021186429 | | 0.171533147 | | 0.171533147 |
| Prevotella_fusca | | I_II | 3.295917688 | 1.403456134 | | 0.021527611 | | 0.171533147 | | 0.171533147 |
| Prevotella_nigrescens | | I_II | 2.213640953 | 0.944508502 | | 0.021789346 | | 0.171533147 | | 0.171533147 |
| Haemophilus_aegyptius | | III | 2.44513544 | 1.019490397 | | 0.023337634 | | 0.179894262 | | 0.179894262 |
| Herbaspirillum_huttiense | | I_II | 1.6368027 | 0.704955334 | | 0.023145696 | | 0.179894262 | | 0.179894262 |
| Aminivibrio_pyruvatiphilus | | I_II | 1.420030062 | 0.614938644 | | 0.023855948 | | 0.181111716 | | 0.181111716 |
| Pseudomonas_aeruginosa | | III | -1.132283872 | 0.489324997 | | 0.023985065 | | 0.181111716 | | 0.181111716 |
| Anaeroglobus_geminatus | | IV | 2.120245749 | 0.92709933 | | 0.025236218 | | 0.186748013 | | 0.186748013 |
| Herbaspirillum_huttiense | | III | 1.535749408 | 0.671923407 | | 0.025146201 | | 0.186748013 | | 0.186748013 |
| Actinomyces_graevenitzii | | IV | -2.490706747 | 1.096485026 | | 0.026012665 | | 0.186887106 | | 0.186887106 |
| Campylobacter_gracilis | | I_II | 1.688028633 | 0.700197642 | | 0.025973997 | | 0.186887106 | | 0.186887106 |
| Peredibacter_starrii | | IV | -0.908591719 | 0.396610461 | | 0.025933527 | | 0.186887106 | | 0.186887106 |
| Sutterella_massiliensis | | IV | 1.66455509 | 0.737791873 | | 0.026976909 | | 0.191951084 | | 0.191951084 |
| Bacteroides_caecigallinarum | | III | -1.557591498 | 0.689151971 | | 0.027318762 | | 0.192532228 | | 0.192532228 |
| Cardiobacterium_valvarum | | I_II | 2.393487196 | 1.066415832 | | 0.027754481 | | 0.193757694 | | 0.193757694 |
| Prevotella_copri | | IV | -0.872948708 | 0.390867315 | | 0.029121195 | | 0.20139892 | | 0.20139892 |
| Abiotrophia_defectiva | | III | 2.300791793 | 1.038702172 | | 0.030972572 | | 0.204955649 | | 0.204955649 |
| Actinomyces_massiliensis | | Benign_tumor | 2.66514962 | 1.208329891 | | 0.030472204 | | 0.204955649 | | 0.204955649 |
| Capnocytophaga_sputigena | | III | 1.999707435 | 0.90511665 | | 0.030203936 | | 0.204955649 | | 0.204955649 |
| Dialister_pneumosintes | | I_II | 1.920359867 | 0.873606788 | | 0.031020315 | | 0.204955649 | | 0.204955649 |
| Prevotella_loescheii | | I_II | 2.577623824 | 1.170654316 | | 0.030750443 | | 0.204955649 | | 0.204955649 |
| Adlercreutzia_muris | | III | -0.676125809 | 0.3093278 | | 0.032669789 | | 0.21394375 | | 0.21394375 |
| Sutterella_massiliensis | | III | 1.436159763 | 0.665740507 | | 0.034269961 | | 0.22245413 | | 0.22245413 |
| Prevotella_maculosa | | I_II | 2.040317291 | 0.949794479 | | 0.034937932 | | 0.224817997 | | 0.224817997 |
| Azospira_oryzae | | IV | -1.174466325 | 0.554246861 | | 0.037779561 | | 0.226453521 | | 0.226453521 |
| Capnocytophaga_granulosa | | I_II | 2.224921246 | 1.05451611 | | 0.038252284 | | 0.226453521 | | 0.226453521 |
| Corynebacterium_matruchotii | | I_II | 2.484333266 | 1.175219962 | | 0.037877497 | | 0.226453521 | | 0.226453521 |
| Corynebacterium_matruchotii | | IV | 2.606701188 | 1.233894298 | | 0.037967275 | | 0.226453521 | | 0.226453521 |
| Delftia_tsuruhatensis | | III | -1.844871639 | 0.863874314 | | 0.036650643 | | 0.226453521 | | 0.226453521 |
| Haemophilus_aegyptius | | IV | 2.602675707 | 1.215201173 | | 0.036923021 | | 0.226453521 | | 0.226453521 |
| Prevotella_buccae | | I_II | 1.7851806 | 0.834342882 | | 0.035638233 | | 0.226453521 | | 0.226453521 |
| Prevotella_saccharolytica | | III | 1.665362912 | 0.77156939 | | 0.036551946 | | 0.226453521 | | 0.226453521 |
| Solobacterium_moorei | | III | -1.229055625 | 0.578661179 | | 0.037852154 | | 0.226453521 | | 0.226453521 |
| Streptococcus_salivarius | | I_II | -4.177528203 | 1.97407752 | | 0.037658032 | | 0.226453521 | | 0.226453521 |
| Lancefieldella_parvula | | I_II | -1.93157569 | 0.920745854 | | 0.039288195 | | 0.230740192 | | 0.230740192 |
| Aliarcobacter_cryaerophilus | | III | -1.096462204 | 0.511800667 | | 0.041113548 | | 0.239559257 | | 0.239559257 |
| Aminivibrio_pyruvatiphilus | | III | 1.183665543 | 0.572704898 | | 0.042947998 | | 0.244438045 | | 0.244438045 |
| Faecalibacterium_prausnitzii | | IV | -1.80943534 | 0.87382264 | | 0.042398091 | | 0.244438045 | | 0.244438045 |
| Leptotrichia_goodfellowii | | IV | 2.311446815 | 1.123776506 | | 0.043177978 | | 0.244438045 | | 0.244438045 |
| Leptotrichia_hofstadii | | I_II | 2.150594184 | 1.046036762 | | 0.04327214 | | 0.244438045 | | 0.244438045 |
| Veillonella_dispar | | III | 0.741943006 | 0.362938474 | | 0.044437737 | | 0.249120645 | | 0.249120645 |
| Prevotella_loescheii | | III | 2.249230422 | 1.107194673 | | 0.045809152 | | 0.254877987 | | 0.254877987 |
| Caldicoprobacter_faecalis | | IV | -1.244405891 | 0.616396703 | | 0.047356227 | | 0.261519463 | | 0.261519463 |
| Capnocytophaga_sputigena | | Benign_tumor | 2.023764146 | 1.011951178 | | 0.049136464 | | 0.269340616 | | 0.269340616 |
| Johnsonella_ignava | | I_II | 1.232077382 | 0.613727671 | | 0.049646219 | | 0.270133839 | | 0.270133839 |
|  | |  |  |  | |  | |  | |  |

**Table S5 Baseline characteristics between the CR/PR group and the SD/PD group.**

|  | CR/PR (n = 14) | SD/PD (n = 46) | P |
| --- | --- | --- | --- |
| Response to chemotherapy |  |  |  |
| CR | 2 |  |  |
| PR | 12 |  |  |
| SD |  | 21 |  |
| PD |  | 25 |  |
| Age (ys, mean ± SD) | 59.86 ± 7.98 | 59.17 ± 11.30 | 0.834 |
| Gender (n, %) |  |  | 0.949 |
| Male | 9 | 30 |  |
| Female | 5 | 16 |  |
| BMI (mean ± SD) | 22.96 ± 2.89 | 22.81 ±3.60 | 0.887 |
| CA19-9 (U/ml, mean ± SD) | 325.29 ± 340.16 | 434.63 ± 391.89 | 0.394 |
| CEA (ng/ml, mean ± SD) | 4.41 ± 5.09 | 8.25 ± 14.50 | 0.397 |
| CHO (mmol/L, mean ± SD) | 4.16 ± 1.29 | 5.14 ± 2.74 | 0.241 |
| LDL (mmol/L, mean ± SD) | 2.42 ± 0.94 | 3.26 ± 2.56 | 0.278 |
| Diabetes |  |  | 0.168 |
| Yes | 6 | 11 |  |
| No  Smoking  Yes  No  Drinking  Yes  No | 8  6  8  6  8 | 35  17  29  20  26 | 0.691  0.967 |

CR/PR, complete response/partial response; SD/PD, stable disease/progressive disease; BMI, body mass index; CA 19-9, carbohydrate antigen 19–9; CEA, carcinoembryonic antigen; CHO, total cholesterol; LDL, low-density lipoprotein cholesterol.

**Table S6 Baseline characteristics between the LTS group and the STS group.**

|  | LTS (n = 28) | STS (n = 35) | P |
| --- | --- | --- | --- |
| Age (ys, mean ± SD) | 57.46 ± 8.14 | 60.40 ± 11.97 | 0.253 |
| Gender (n, %) |  |  | 0.682 |
| Male | 17 | 23 |  |
| Female | 11 | 12 |  |
| BMI (mean ± SD) | 23.49 ± 2.50 | 21.98 ±3.48 | 0.059 |
| CA19-9 (U/ml, mean ± SD) | 320.24 ± 354.38 | 464.95 ± 393.04 | 0.190 |
| CEA (ng/ml, mean ± SD) | 8.54 ± 16.28 | 9.12 ± 17.09 | 0.906 |
| CHO (mmol/L, mean ± SD) | 4.30 ± 1.15 | 5.52 ± 3.17 | 0.082 |
| LDL (mmol/L, mean ± SD) | 2.57 ± 0.90 | 3.54 ± 2.99 | 0.134 |
| Diabetes |  |  | 0.759 |
| Yes | 9 | 10 |  |
| No  Smoking  Yes  No  Drinking  Yes  No | 19  10  18  12  16 | 25  14  21  17  18 | 0.728  0.651 |

LTS, long term survival; STS, short term survival; BMI, body mass index; CA 19-9, carbohydrate antigen 19–9; CEA, carcinoembryonic antigen; CHO, total cholesterol; LDL, low-density lipoprotein cholesterol.
